# Rational design of phase separating peptides based on phase separating protein sequence of p53

**DOI:** 10.1038/s41598-023-32632-2

**Published:** 2023-04-06

**Authors:** Kiyoto Kamagata, Atsumi Hando, Maulana Ariefai, Nanako Iwaki, Saori Kanbayashi, Ryotaro Koike, Keisuke Ikeda

**Affiliations:** 1 Institute of Multidisciplinary Research for Advanced Materials, Tohoku University, Katahira 2-1-1, Aoba-ku, Sendai, 980-8577 Japan; 2 Graduate School of Life Sciences, Tohoku University, Sendai, 980-8578 Japan; 3 Department of Chemistry, Faculty of Science, Tohoku University, Sendai, 980-8578 Japan; 4 Department of Chemistry, Graduate School of Science, Tohoku University, Sendai, 980-8578 Japan; 5 Graduate School of Informatics, Nagoya University, Nagoya, Aichi 464-8601 Japan; 6 Department of Biointerface Chemistry, Faculty of Pharmaceutical Sciences, University of Toyama, 2630 Sugitani, Toyama, 930-0194 Japan

**Keywords:** Peptides, Proteins, Biomaterials - proteins, Computational biophysics, Intrinsically disordered proteins, Single-molecule biophysics, Fluorescence imaging

## Abstract

Artificial phase-separating (PS) peptides can be used in various applications such as microreactors and drug delivery; however, the design of artificial PS peptides remains a challenge. This can be attributed to the limitation of PS-relevant residues that drive phase separation by interactions of their pairs in short peptides and the difficulty in the design involving interaction with target PS proteins. In this study, we propose a rational method to design artificial PS peptides that satisfy the requirements of liquid droplet formation and co-phase separation with target PS proteins based on the target PS protein sequence. As a proof of concept, we designed five artificial peptides from the model PS protein p53 using this method and confirmed their PS properties using differential interference contrast and fluorescence microscopy. Single-molecule fluorescent tracking demonstrated rapid diffusion of the designed peptides in their droplets compared to that of p53 in p53 droplets. In addition, size-dependent uptake of p53 oligomers was observed in the designed peptide droplets. Large oligomers were excluded from the droplet voids and localized on the droplet surface. The uptake of high-order p53 oligomers into the droplets was enhanced by the elongated linker of the designed peptides. Furthermore, we found that the designed peptide droplets recruited p53 to suppress gel-like aggregate formation. Finally, we discuss aspects that were crucial in the successful design of the artificial PS peptides.

## Introduction

Liquid–liquid phase separation (LLPS) can concentrate molecules with various unique biological functions in membraneless organelles at levels that cannot be obtained in the dilute bulk phase^1–5^. Various proteins, including FUS^6^, LAF-1^7^, and p53^8^ can form liquid droplets via LLPS. Liquid droplets are stabilized by multivalent intermolecular interactions between the amino acid pairs of intrinsically disordered regions (IDRs) of proteins^6,8–14^. Such self-LLPS of these proteins is categorized into simple associative coacervation, rather than complex or segregative one. In addition, artificial phase separating (PS) tools have been produced by combining or repeating natural protein sequences with a linker spacer^15–19^. Although successful examples of long PS sequence design are known, artificial PS peptide design remains challenging. In particular, single-component associative PS peptides are limited. However, many peptide-peptide complex coacervates have been reported^20^. Artificial PS peptides can be used for various applications, such as the development of co-phase separation for storing target proteins, microreactors for catalytic reactions^21^, and therapeutics^22^.

Three approaches have been used to discover and produce single-component associative PS peptides. In the first approach, PS peptides are discovered by searching for strongly self-interacting peptide fragments in PS proteins. A LLPS-relevant peptide fragment of 23 residues was identified in the IDR of the histidine-rich squid beak protein HBP-1^23^. In addition, low-complexity aromatic-rich kinked segments (LARKS) or reversible amyloid cores (RAC) that form reversible hydrogels or amyloids have been found in stress granule-associated proteins (FUS and hnRNPA1) and porin nup98^24–26^. In the second approach, a PS peptide with 25 residues was produced from the mussel adhesive protein mfp-3S (45 residues) by selectively removing residues that are not strongly involved in LLPS^27^. In the third approach, many dipeptides were tested, and they formed liquid droplets or solid aggregates^21,28^.

The limited number of single-component associative PS peptides compared to the amount of PS proteins might be attributed to the lack of a theoretical design framework. Typically, PS proteins have many residues that can interact relatively-strongly to the same or other residues (R, K, D, E, Y, and F). These residues are dispersed throughout the sequences, resulting in the formation of liquid droplets via multivalent but weak interactions. In this study, we call the residues that are involved in electrostatic, caiton-π, and π–π interactions driving LLPS as PS residues (R, K, D, E, Y, F, and W). Note that PS residues cannot undergo LLPS by themselves and multivalent interactions between PS residue pairs such as R and D are required for the LLPS. In contrast to the multivalent interaction of PS proteins, most of the peptide fragments derived from PS proteins, except for LARKS and RAC, would not undergo phase separation because a relatively weak self-interaction is expected due to the limited number of PS residues. Hence, PS peptides should be designed to possess many PS residues. In addition, considering the application of a liquid droplet that can recruit PS proteins, the PS peptide should be designed to interact with the target PS proteins.

**Figure 1:**
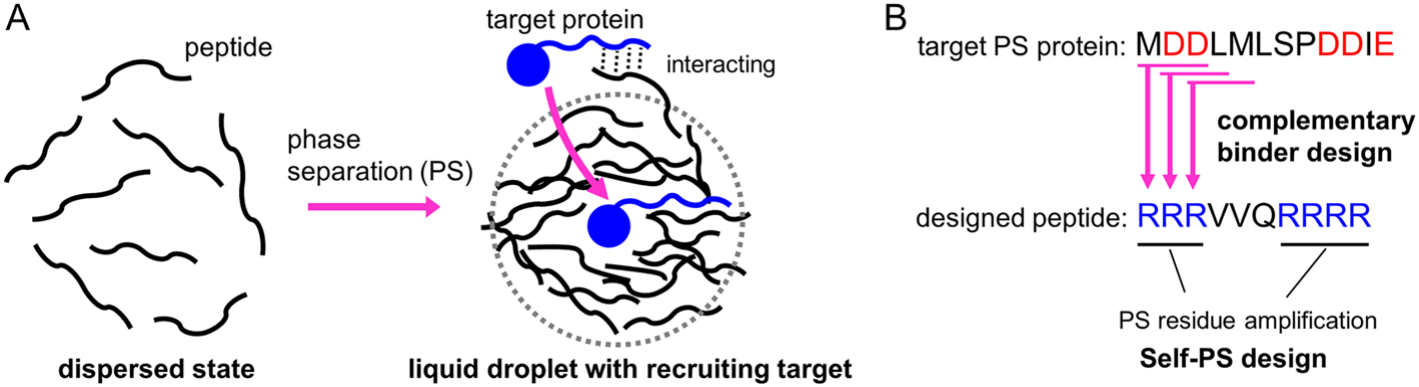
Design concept of artificial PS peptide. (**A**) Liquid droplet formation of PS peptide and recruitment of a target protein. Peptides (black) dispersed in solution form a liquid droplet, and the target protein (blue) is recruited into the droplet. (**B**) Scheme of one-by-three design targeting PS protein sequence (pink arrow). This provides the complementary peptide binder sequence and PS residue amplification in the peptide (e.g. 7 R in peptide vs. 4 D plus 1 E in target) for self-PS. p53 N-terminal IDR sequence is displayed as an example of target PS protein. Red and blue characters of sequences correspond to PS residues.

To design artificial associative PS peptides that satisfy the two requirements of liquid droplet formation and recruitment of target PS proteins into the droplets, we speculated that our theoretical peptide binder design method could be utilized (Fig. 1). This method was originally developed to computationally design artificial peptides that can bind to the target IDR based on the residue-residue contact free energy^29,30^. Using this method, the peptides designed to target IDRs of the model protein p53 functioned as regulators of its DNA-binding affinity^29^ and liquid droplet formation^30^. To form droplets having co-phase separation ability with target PS proteins, peptides designed from the targets need to bind to PS proteins, thereby assisting with their uptake into the droplets. The peptide binder design can be used for this purpose. In addition, taking into account neighbor residue interactions, we noticed that the one-by-three design for PS protein fragments amplified PS residues in the designed peptides via processes such as charged residue amplification^29,30^. Briefly, the one-by-three design predicts the residue that strongly interacts to three sequential residues of target (minimizing the sum of contact energies against the three residues) (Fig. 1B). For example, if target contains one negatively-charged residue, two or three positively-charged residues are predicted in the peptide binder. In the peptide binder, such charged or aromatic residues tend to appear sequentially, and these residue numbers are increased or amplified compared to those of the target sequence. In particular, clusters (consecutive repeats) of charged or aromatic residues in the designed peptides are expected to promote PS ability, as reported previously^21,31–35^. Accordingly, peptides designed via the one-by-three strategy seemed to undergo phase separation.

In this study, we designed artificial associative PS peptides from a model PS protein p53 using the theoretical peptide binder design method and tested their phase separation and target-recruiting capabilities. Our results highlight the designability of the artificial PS peptide droplets.

## Results

### Designing PS peptides based only on the sequence of the PS protein

**Figure 2:**
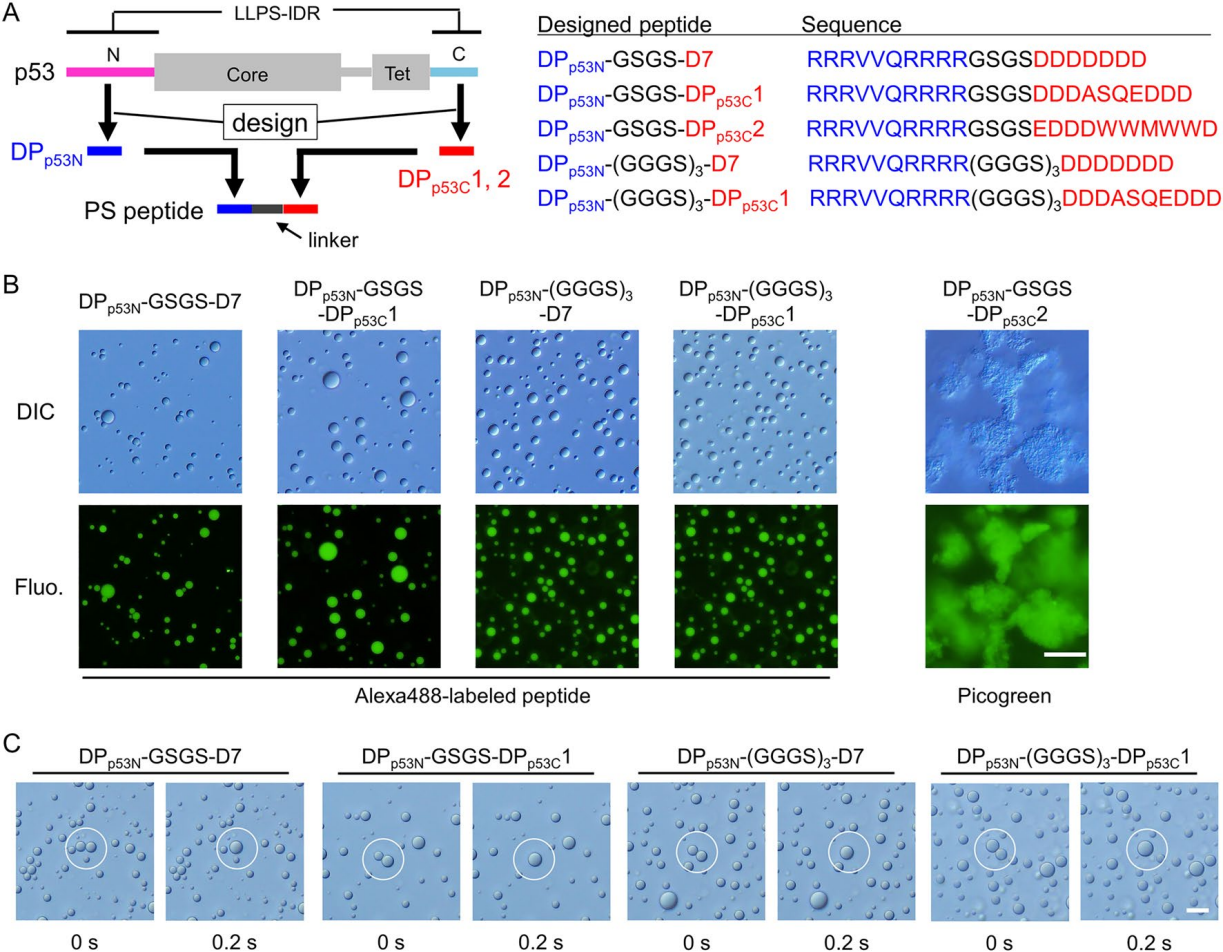
Design scheme and experimental verification of artificial PS peptides from PS protein p53. (**A**) The design scheme and peptide sequences designed based on the p53 sequence. N, core, Tet, and C represent the N-terminal, core, tetramerization, and C-terminal domains of p53, respectively. Large boxes represent folded regions, whereas thin lines represent disordered regions. Complementary peptide fragments (blue and red) were designed based on two LLPS-relevant IDRs (pink and light blue) of p53 and were then connected by a linker (black). (**B**) DIC and fluorescence images of the solution containing designed peptides in the concentration range of 0.9–1.8 mM in the absence of salt at pH 7.0. Alexa488-labeled peptide at 0.1 μM was added to confirm high concentrations in liquid droplets. Picogreen was used as an amyloid-sensitive probe. (**C**) Typical fusion snapshots of two droplets (white circles) in the solution containing 1 mM designed peptide in the absence of salt at pH 7.0. Scale bars in panels B and C denote 20 and 10 μm, respectively.

We designed five PS peptide candidates based on two PS-relevant IDRs (N-terminal and C-terminal domains) of p53 that were identified to form liquid droplets^8^ (Fig. 2A). DP_p53N_ was complementary binder for p53 N-terminal domain (residues 41–50) which was designed using molecular dynamics (MD)-based contact energy with one-by-three interactions^30^. Note that the MD-based contact energy would be better than the 3D-based contact energy proposed by Miyazawa and Jernigan because the former reflects residue-residue interactions while minimizing backbone constraints^30^. As interaction partners of DP_p53N_, complementary binders for the C-terminal domain of p53, DP_p53C_1 and DP_p53C_2, were designed using a same method^30^ (see “Materials and methods”). The two complementary peptides were connected via a GSGS linker to produce intermolecular interactions (DP_p53N_-GSGS-DP_p53C_1 and DP_p53N_-GSGS-DP_p53C_2). This step devised in this study is key for producing the PS peptide candidates: many PS residue pairs between the complementary peptides (e.g. R and D pair or R and W pair) are required to interact each other. The other point is to reduce the absolute net charge of the PS peptide candidates to ~ 0 for minimizing the electrostatic repulsion. In addition, we prepared an alternative PS peptide candidate, DP_p53N_-GSGS-D7, in which D7 was added to reduce the intermolecular electrostatic repulsion by canceling the positive charges of DP_p53N_ and interacting with DP_p53N_ of other molecules electrostatically. Furthermore, two PS peptide candidates, DP_p53N_-(GGGS)_3_-DP_p53C_1 and DP_p53N_-(GGGS)_3_-D7, were designed to test the effect of the linker length. Sequence analysis of the PS peptide candidates and natural proteins revealed that the maximum sequence identities ranged from 41 to 58% for the full-length designed peptides, but were at least 70% for the complementary binder peptides (Supplementary Table S1).

Differential interference contrast (DIC) microscopy confirmed that these peptides (0.9–1.8 mM) underwent phase separation in 25 mM HEPES and 0.5 mM EDTA at pH 7.0 (Fig. 2B). To prevent the melting of clusters on the coverslip surface, 2-methacryloyloxyethyl phosphorylcholine (MPC) polymer coating was used^36,37^ (Supplementary Fig. S1). Samples were prepared by sonicating a 2 mM peptide solution or diluting the peptide solution from a 10 mM peptide stock after storage under freezing conditions. Four of the designed peptides, except DP_p53N_-GSGS-DP_p53C_2, formed μm-sized spherical clusters. Fluorescence microscopy confirmed the high concentration of peptides labeled with the fluorescent dye Alexa488 in the clusters (Fig. 2B). The fusion of two clusters was observed for the four designed peptides within 0.2 s, indicating the liquid droplet property (Fig. 2C). In contrast, DP_p53N_-GSGS-DP_p53C_2 formed non-spherical clusters that corresponded to solid aggregates (Fig. 2B). The amyloid-sensitive probe, Picogreen^38,39^, exhibited a high intensity for the clusters of DP_p53N_-GSGS-DP_p53C_2, implying the formation of a cross-β structure in the aggregates (Fig. 2B). Cross-β structure was also supported by the observation of intrinsic fluorescence upon 400–440 nm excitation^40^ (Supplementary Fig. S2). Clusters of aromatic W residues in DP_p53N_-GSGS-DP_p53C_2 may lead to the formation of solid aggregates, which is consistent with the results of different systems^21,34^. The formation of aggregates was not significantly suppressed at high concentrations of salt and 1,6-hexanediol^13^, suggesting that solid aggregates were stabilized by the cross-β structure rather than electrostatic or non-electrostatic interactions (Supplementary Fig. S3). Hence, we proposed a method for designing PS peptides based on natural PS protein sequences and demonstrated the phase separation of five peptides designed based on the p53 sequence.

### Differences in droplet characteristics between p53-based designed peptides and p53

**Figure 3:**
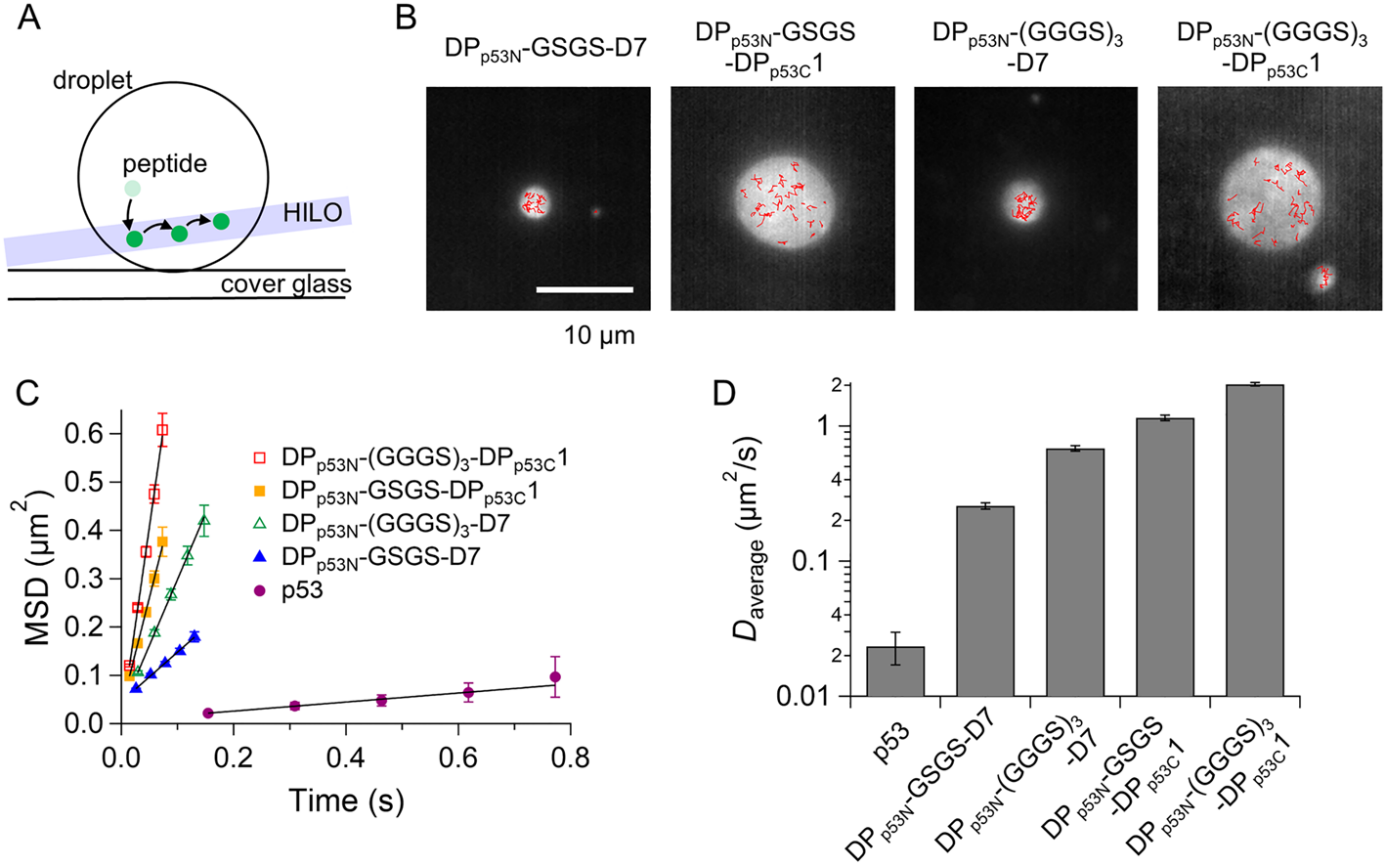
Diffusion dynamics of designed peptide and p53 in their droplets. (**A**) Schematic diagram of single-molecule tracking of fluorescent peptides or p53 in a droplet. HILO illumination minimizes background fluorescence, enabling single-molecule detection of labeled peptides (green) in droplets. (**B**) Typical trajectories of Alexa488-labeled designed peptides in corresponding droplets in 45 mM NaCl and 150 mg/mL dextran at pH 7.0. The typical trajectories of single molecules (red) are overlaid in fluorescent images derived based on time averaging (white droplets). (**C**) Mean square displacement plots of Alexa488-labeled designed peptides or Atto488-labeled p53 in corresponding droplets. The straight lines show the best-fit linear functions for the MSD data. Error bars denote standard errors. (**D**) Comparison of diffusion coefficients of the designed peptides and p53 in corresponding droplets.

We quantitatively characterized the liquid droplet properties of the four designed peptides and compared them with those of p53. We measured the fusion of the designed peptide droplets in the solution used for p53 droplets (Supplementary Fig. S4). In the presence of 45 mM NaCl and 150 mg/mL dextran, the fusion of droplets for the four designed peptides was observed within 0.2 s, which was much faster than that for p53 (3.7 min)^8^. This gap in the fusion period implies different intrinsic dynamic properties. We measured the movement of Alexa488-labeled peptides within non-labeled peptide droplets using single-molecule fluorescence microscopy with a highly inclined and laminated optical sheet (HILO) illumination^37,41^ (Fig. 3A). Figure 3B shows the trajectories of the labeled peptides plotted on the droplet in 45 mM NaCl and 150 mg/mL dextran. The mean square displacement (MSD) plots of the trajectories demonstrated a linear relationship with time, indicating that the four designed peptides diffused inside the droplets (Fig. 3C). The average diffusion coefficient (*D*) obtained by fitting the MSD plots with a linear equation with a 4*D* slope ranged from 0.26 ± 0.01 μm^2^/s for DP_p53N_-GSGS-D7 to 2.03 ± 0.06 μm^2^/s for DP_p53N_-(GGGS)_3_-DP_p53C_1 (Fig. 3D). The *D* values of the designed peptides were 11-to 87-fold larger than that of p53 in p53 droplets, consistent with the faster fusion of droplets observed with the designed peptides compared to that of p53 (Fig. 3D and Supplementary Fig. S4). This rapid diffusion of the designed peptides might reflect a relatively weak intermolecular interaction compared to that of p53, because the p53 tetramer has eight sets of droplet-forming IDRs (N-terminal and C-terminal IDRs) along with a large number of PS residues (140 per tetramer). Note that the diffusion of D7 peptides was slower than that of DP_p53C_1 peptides, consistent with the two-fold higher affinity of the DP_p53N_ fragment for the D7 fragment than that for the DP_p53C_1 fragment (Supplementary Fig. S5). The relatively weak interaction of designed peptides is consistent with the fact that the critical concentrations of designed peptides (100–200 µM) are 100–200-fold larger than that of p53 (Supplementary Fig. S6A).

We investigated the intermolecular interactions of peptides within the droplets. Since the designed peptides possess 14 charged residues (seven positively and seven negatively charged), electrostatic interactions might mediate droplet formation. An increase in the salt concentration decreased light scattering observed with the solution of the four designed peptides at 350 nm (Supplementary Fig. S7A). DIC microscopy confirmed that the number of droplets decreased with increasing salt concentrations (Supplementary Fig. S7B). The lower tolerability of DP_p53C_1 peptides to salt concentration compared to D7 peptides is consistent with the weak affinity of the DP_p53C_1 fragment for the DP_p53N_ fragment (Supplementary Fig. S5). In contrast, 1,6-hexanediol, which weakens non-electrostatic interactions, did not affect light scattering attributed to the designed peptide droplets and their DIC images (Supplementary Fig. S7C, D). In addition, the fluorescence intensity of Picogreen in droplets was higher than that in the solution for DP_p53N_-(GGGS)_3_-DP_p53C_1, whereas that in droplets was comparable to or slightly higher than that in the solution for the other designed peptides (Supplementary Fig. S8). Similarly, DP_p53N_-(GGGS)_3_-DP_p53C_1 in droplets showed high intrinsic fluorescence (Supplementary Fig. S2). These results imply the formation of a cross-β structure in the droplets of DP_p53N_-(GGGS)_3_-DP_p53C_1; however, the formation was not significant for the droplets of other designed peptides. Note that the four designed peptides are in random coil states under no PS condition (Supplementary Fig. S6B). Taken together, electrostatic interactions are the key determinant for the formation of liquid droplets for the designed peptides, similar to p53 droplets, except for the 1,6-hexanediol dependence^8^.

### Moderate-selective uptake of peptides into designed peptide droplets

Since various guest proteins are recruited into droplets of natural PS protein p53^37^, we next investigated the recruitment specificity of guest peptides into droplets of PS peptides (Supplementary Fig. S9A). The fluorescence images demonstrated that the N-terminal peptide of p53 was highly localized inside the droplets of GSGS-linker peptides compared to that of the C-terminal peptide of p53 (Supplementary Fig. S9B). For quantitative comparison, we used the average enrichment index (EI) calculated as the ratio of the fluorescence intensity in droplets to that in solution (Supplementary Fig. S9C). The average EI of the N-terminal peptide of p53 was 1.8–3.7-fold larger than that of the C-terminal peptide of p53, indicating the high uptake of the N-terminal peptide rather than the C-terminal peptide. We next examined the sequence specificity of uptake by comparing the EI values of N-terminal peptide of p53 and of its sequence-shuffled peptides in DP_p53N_-GSGS-D7 droplet. The average EI of the N-terminal shuffled peptides of p53 were reduced by 1.4–1.9-fold from that of the N-terminal peptide of p53, suggesting the moderate significance of guest peptide sequences on the uptake (Supplementary Fig. S9D). In addition, the uptake level of the peptide fragment from other PS protein FUS was lower than that of the N-terminal peptide of p53 and was comparable to that of the N-terminal shuffled or C-terminal peptides of p53 (Supplementary Fig. S9D). These results demonstrate moderately-selective uptake of guest peptides into peptide droplets as observed for the molecular uptake of p53 droplets.

### Size-dependent uptake of p53 oligomers into designed peptide droplets and enhanced uptake of high-order oligomers mediated by an elongated linker

We tested whether the designed peptides originating from p53 functioned as droplets with uptake of p53. As a monomer, p53 contains 393 residues (a residue length that is at least 12-fold larger than that of the designed peptides) and forms a tetramer. If the molecular size of p53 is larger than the droplet void sizes, p53 molecules should be excluded from the droplets in a size-dependent manner^16,41–43^. Considering this, we examined the uptake properties of Alexa488-labeled p53 and its different oligomeric mutants in the droplets of non-labeled designed peptides with a GSGS linker.

**Figure 4:**
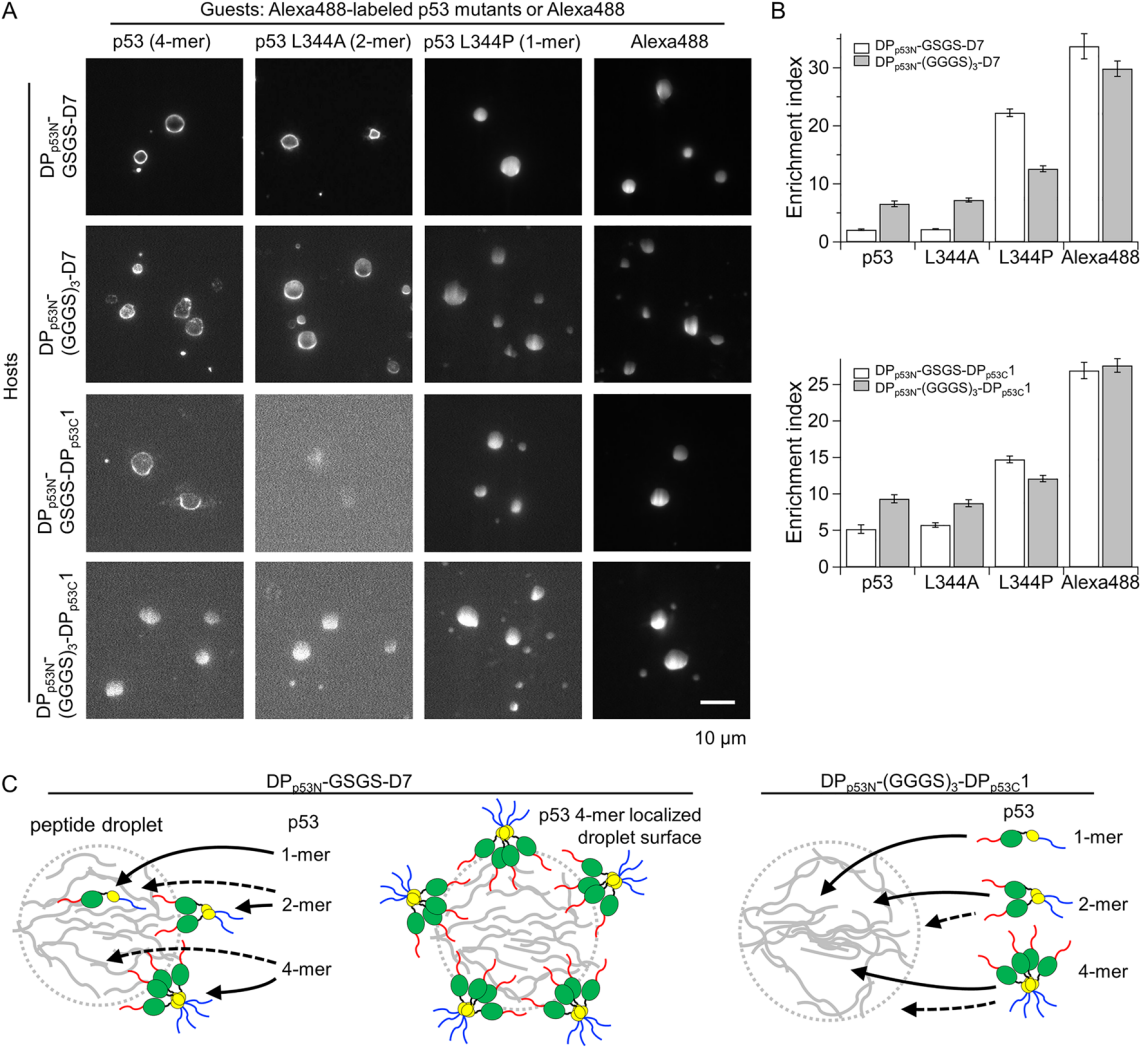
Size-dependent uptake of p53 oligomers into designed peptide droplets and enhanced uptake of high-order oligomers upon elongation of the linker. (**A**) Fluorescence images of Alexa488-labeled p53 oligomers and Alexa488 in non-labeled designed peptide droplet solution. The solution contained designed peptides with the concentration range of 0.9–1.8 mM and 0.1 µM of labeled p53 oligomers or Alexa488 in the absence of salt at pH 7.0. (**B**) Enrichment index of Alexa488-labeled p53 oligomers and Alexa488 in the designed peptide droplets. Error bars denote the standard errors. (**C**) Proposed models of size-dependent uptake of p53 oligomers into the droplets of two representative designed peptides with GSGS- and (GGGS)_3_-linkers. The droplet (dashed grey circle) is formed by designed peptides (grey). As shown in the left panel, uptake of p53 dimer and tetramer was limited compared to that of p53 monomer, likely owing to the small droplet voids of GSGS-linker peptides. As shown in the middle panel, p53 tetramers may be localized onto the droplet surface due to their exclusion from droplets and interaction between designed peptides and p53. As shown in the right panel, the uptake of p53 dimer and tetramer into (GGGS)_3_-linker peptide droplets was enhanced since the linker elongation seemed to increase droplet voids. Solid and dashed arrows represent the major and minor events for the uptake inside the droplets or the localization on droplet surfaces, respectively. Red, green, black, yellow, and blue represent N-terminal IDR, core domain, linker, tetramerization domain, and C-terminal IDR of p53, respectively.

The fluorescence images demonstrated that the p53 monomer (L344P) mutant was highly localized inside the droplets of GSGS-linker peptides, similar to Alexa488 only (Fig. 4A). In contrast, the p53 dimer (L344A) mutant and tetramer were localized on the droplet surfaces of GSGS-linker peptides, except in the case of the p53 dimer in the DP_p53N_-GSGS-DP_p53C_1 droplet, and had a low recruitment tendency inside the droplets compared to the monomer mutant (Fig. 4A). The EI of the p53 monomer (L344P) mutant was 22.3 ± 0.7 for DP_p53N_-GSGS-D7 and 14.7 ± 0.5 for DP_p53N_-GSGS-DP_p53C_1 (Fig. 4B). In contrast, the average EI values of the p53 tetramer, which reflect droplet interior regions but do not include the droplet surface, decreased by 10.5-fold for DP_p53N_-GSGS-D7 and by 2.6-fold for DP_p53N_-GSGS-DP_p53C_1 (Fig. 4B). These results clearly demonstrate size-dependent recruitment and exclusion of p53 proteins. Considering that the EI values of the p53 dimer were similar to those of the p53 tetramer, the void size of the GSGS-linker peptide droplets ranged between those of the p53 monomer (393 residues) and dimer (786 residues). Therefore, the p53 monomer was inserted into the voids of the GSGS-linker peptide droplets, whereas the p53 dimer and tetramer were largely excluded from the voids and localized onto their droplet surfaces (Fig. 4C). The high recruitment of the N-terminal peptide of p53 compared to that of the C-terminal peptide of p53 implied that the N-terminal IDR of p53, rather than the C-terminal IDR, interacted with the designed peptides inside the droplets or on droplet surfaces (Supplementary Fig. S9C). Similarly, the localization on the droplet surfaces of DP_p53N_-GSGS-D7 was observed for other guest protein, maltose binding protein-fused FUS (FUS-MBP; 934 residues) (Supplementary Fig. S10). The average EI of FUS-MBP was 2.2-fold smaller than that of the p53 monomer. The size-dependent exclusion of p53 oligomers from droplets and their localization on their surfaces are unique to the designed peptides; these properties have not been observed in the droplets of the p53 tetramer^37^.

If the void sizes of the designed peptide droplets are determined by the peptide length, elongation of the linker could promote the uptake of the p53 dimer and tetramer. To test this hypothesis, we measured the uptake of p53 oligomers into the droplets of (GGGS)_3_-linker peptides (Fig. 4A). The average EIs of the p53 dimer mutant increased by 3.3-fold for DP_p53N_-(GGGS)_3_-D7 and 1.5-fold for DP_p53N_-(GGGS)_3_-DP_p53C_1 compared to those of their GSGS-linker peptides (Fig. 4B). Similarly, the average EIs of the p53 tetramer increased by 3.1-fold for DP_p53N_-(GGGS)_3_-D7 and 1.8-fold for DP_p53N_-(GGGS)_3_-DP_p53C_1 compared to those of their GSGS-linker peptides (Fig. 4B). For DP_p53N_-(GGGS)_3_-DP_p53C_1, the surface localization of the p53 dimer mutant and tetramer was not observed, unlike other designed peptides, supporting the enhanced uptake inside the droplets. In contrast, the average EIs of the p53 monomer mutant decreased by 1.8-fold for DP_p53N_-(GGGS)_3_-D7 and by 1.2-fold for DP_p53N_-(GGGS)_3_-DP_p53C_1 compared to those for their GSGS-linker peptides, which may indicate that an appropriate void size is required for high recruitment (Fig. 4B). Taken together, the elongation of the linker of the designed peptides enhanced the uptake of high-order oligomers of p53, likely by increasing the void size of their droplets (Fig. 4C).

### Recruitment of p53 into the designed peptide droplets suppressed the formation of gel-like aggregates of p53

**Figure 5:**
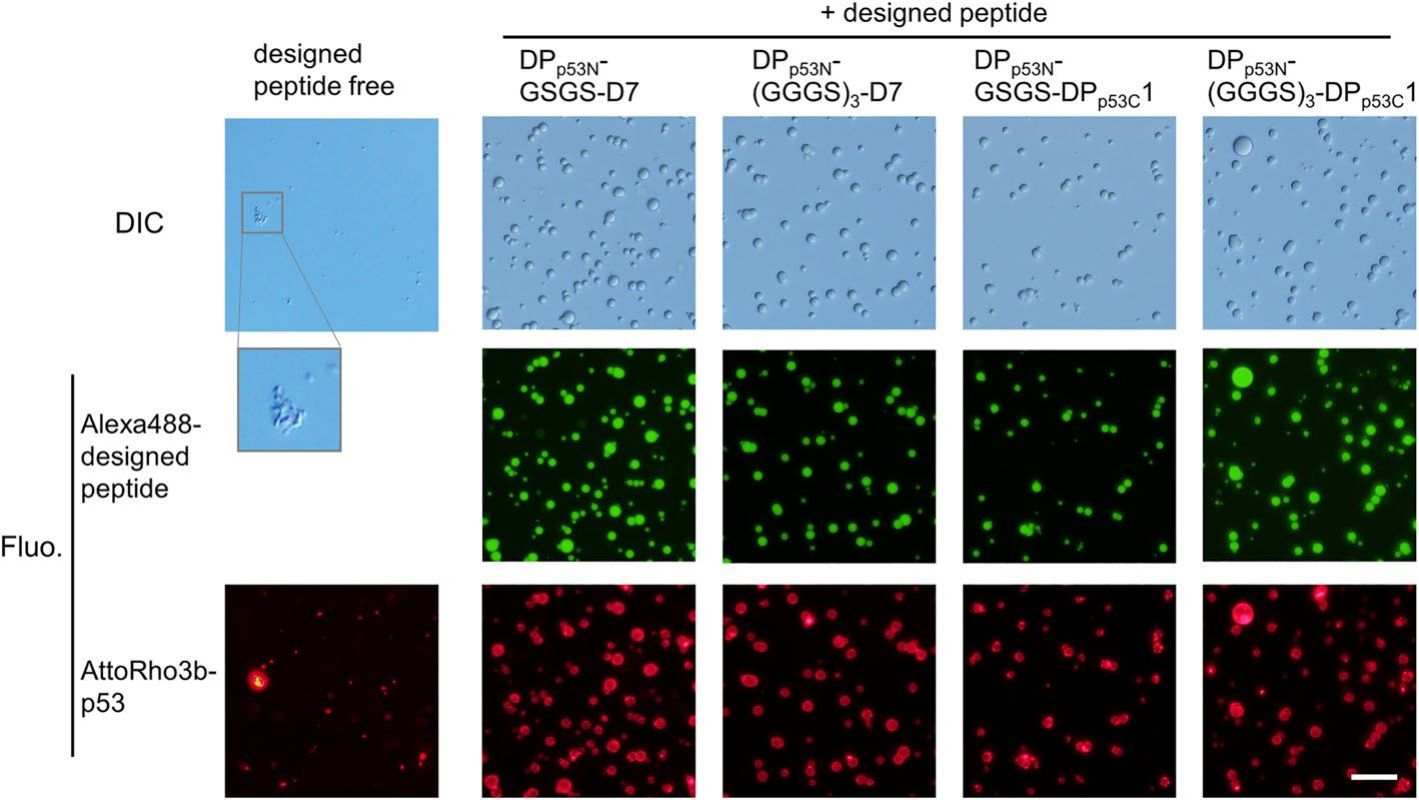
Recruitment of p53 into designed peptide droplets suppressed the formation of gel-like aggregates of p53. DIC and fluorescence images of the solution containing 1 mM designed peptides and 1.25 µM p53 (tetramer) in 45 mM NaCl at pH 5.5. For visualizing the localization, alexa488-labeled designed peptide and AttoRho3b-labeled p53 were added at a concentration of 0.1 µM. The scale bar denotes 20 μm. A region labeled with an ocher square has been enlarged.

We next tested the co-phase separating function of the designed peptide droplets under conditions in which p53 itself could form gel-like aggregates in 45 mM NaCl at pH 5.5^8^ (Fig. 5). When we incubated 1 mM designed peptides and 1.25 µM p53 (tetramer) under these conditions (47–61 of the mass ratio of designed peptides against p53), spherical droplets or their assemblies were observed in the DIC images. In contrast, nonspherical gel-like clusters of p53 were observed in the absence of the designed peptides. The fluorescent images indicated that p53 molecules were recruited into the designed peptide droplets and dispersed uniformly inside the designed peptide droplets along with the localization on the droplet surface to some extent, indicating that the designed peptide droplets suppressed the formation of gel-like assemblies of p53 itself. In addition, we observed the fusion events of the two droplets containing p53, confirming the fluidic properties of the droplets (Supplementary Fig. S11). Note that the fusion time of the droplets increased upon co-phase separation with p53. In contrast, when p53 concentration was increased by tenfold (12.5 µM) corresponding to 4.7–6.1 of the mass ratio of designed peptides against p53, non-spherical gel-like clusters containing the designed peptides and p53 were observed (Supplementary Fig. S12). Under these conditions, the designed peptide droplets could not prevent the formation of gel-like aggregates of p53 molecules. Accordingly, the recruitment of p53 into designed peptide droplets function with at least ~ 47 of the mass ratio of p53 against designed peptides.

### Validation of design method for other PS protein

We tested the designability of the artificial PS peptide droplets for other PS protein FUS using the theoretical peptide binder design method (Supplementary Fig. S13A). FUS droplets are stabilized via cation–π and hydrophobic interactions^6,13,39,44^, which are different from the electrostatic interactions in p53 droplets^8^. DIC and fluorescence images confirmed that the designed peptide of FUS formed liquid droplets and the peptide concentration inside the droplets was high (Supplementary Fig. S13B). Furthermore, the designed peptide of FUS showed the recruitment of FUS-MBP into the droplets while highly localizing the droplet surfaces (EI = 9.6 ± 0.3; Supplementary Fig. S13C). These results highlight the designability of peptides with self-PS and target recruitment capability.

## Discussion

Our design method for artificial PS peptides was unique in terms of the production of artificial peptides that possess both self-PS and target-uptake properties. One of the previously proposed methods involves searching for strongly self-interacting peptide fragments over PS protein sequences. Although several PS peptides have been identified^23–26^, the use of this method is limited to natural PS proteins possessing a short fragment with a strong PS ability. In contrast, our method involved the production of peptide sequences with amplified PS residues, as shown with the example of p53 as well as FUS. Another proposed method involves shortening PS protein sequences by selectively removing residues that are not strongly involved in LLPS^27^. The enhancement of the relative fraction of PS residues upon peptide shortening was similar to that was taken into account in our method. However, our method enabled the incorporation of the property facilitating the uptake of the target PS protein along with their PS property.

The experimental verification of our artificial PS peptides demonstrated some points that contributed to their successful design. The designed peptides showed physical characteristics similar to or different from those of the target PS protein. Liquid droplet formation of the designed peptides was driven by electrostatic interactions but not hydrophobic interactions (Supplementary Fig. S7). This reflects that the properties of p53 IDRs are relevant to LLPS, which possesses many charged residues^8^. The residues complementary to the charged residues are designed as oppositely charged residues, and their numbers in the designed peptides are amplified via a one-by-three design^30^. The charged residue clusters in the designed peptides may contribute to enhance LLPS activity, as reported elsewhere^31–33,35^. In general, our design methods tend to select PS-relevant residues as complementary residues against PS residues in targets and produce their clusters, because a PS residue shows relatively high free energy for interaction with same or other interaction-partner PS residues^30^. The PS peptides designed based on p53 sequence include many Arg and Asp residues rather than Lys and Glu residues, which are consistent with the fact that complex coacervation of Arg- and Asp-polymer pairs showed high tolerance against salt compared to that of other charged residue polymer pairs^45^. The absence of β-hairpin backbone structure of designed peptides under no PS condition might not block the conversion from intramolecular interactions between oppositely-charged side chains in the monomer to intermolecular interactions formed in the droplets (Supplementary Fig. S6B). In contrast, the critical concentration of designed peptides was much higher than that of p53. This might attribute to the high stability of p53 droplets via the large total number of PS residues and tetrameric structure.

In DP_p53N_-GSGS-DP_p53C_2, the aromatic tryptophan clusters facilitated solid aggregation via cross-β structure formation (Fig. 2B). WW dipeptides are known to form solid aggregates^21^. This is also supported by the fact that the aromatic patches in the low-complexity domain of hnRNPA1 aided in the conversion of liquid droplets into solid aggregates^34^. For DP_p53N_-(GGGS)_3_-DP_p53C_1, long-term incubation enhanced the cross-β structures in droplets, resulting in gel-like assemblies of the droplets. Accordingly, it is necessary to avoid the clustering of cross-β-structure forming residues, such as aromatic residues, in designed peptides for producing liquid droplets, whereas it would be acceptable to produce a cluster of charged residues.

Molecular uptake into the designed peptide droplets can be tuned by modifying the linker length and/or intermolecular interactions of the designed peptides. Linker elongation would be useful to promote the molecular uptake of relatively large molecules. In fact, linker elongation by threefold enhanced the molecular uptake of the p53 dimer and tetramer by 1.5–3.3-fold (Fig. 4B). The elongation between PS-residue clusters in the designed peptides seems to increase the void volumes inside the droplets, reducing the size exclusion of relatively large molecules. The presence of voids was suggested in the droplets of several PS proteins^16,41,46,47^. In addition, the weakening of intermolecular interactions is expected to enhance the void volumes. The uptake of the p53 dimer and tetramer in DP_p53C_1-peptide droplets was 2.3–2.7-fold higher than that in D7-peptide droplets (Fig. 4B). This was attributed to the twofold weaker intermolecular interaction of the DP_p53C_1 fragment with the DP_p53N_ fragment compared to that of the D7 fragment (Supplementary Fig. S5). In contrast, the designed peptides with short linker and strong intermolecular interactions could reduce the void size, which is effective for the selective uptake of relatively small targets (e.g., the p53 monomer in this study). The relatively slow translational dynamics observed in DP_p53N_-GSGS-D7 may stabilize small voids.

## Conclusion

We provide a design method for artificial peptides that undergo phase separation by themselves and recruit target PS proteins inside their liquid droplets based on a natural PS protein sequence. As a proof of concept, we confirmed that the PS peptides, designed from a model PS protein p53, underwent LLPS, and p53 was recruited into their droplets. Also, the design method worked for the other model PS protein FUS. Accordingly, this method could be applied to other PS proteins to produce co-phase separation with PS peptides for various purposes.

## Materials and methods

### Peptide design

For the N-terminal IDR of p53, we used the designed peptide (DP_p53N_) reported in our previous study^30^. For the C-terminal IDR of p53, peptides were designed as described in our previous studies^29,30^. Briefly, the relative binding free energy between the *i*th residue of the C-terminal IDR and the *j*th residue of the designed peptide was calculated as *e*_*ij*_ + *e*_*rr*_ − *e*_*ir*_ − *e*_*jr*_, where *e*_*ij*_ denotes the binding free energy between the *i*th and *j*th residues^30^, and *r* represents the average amino acid residue, as defined by Miyazawa and Jernigan^48^. We considered the energy derived from the MD-based analysis of the side chains^30^. We calculated the binding free energy by replacing the *j*th residue of a designed peptide with each of the 20 residues and then determined the residues with the lowest binding free energy. We used a one-by-three design by adding the binding energy between adjacent residues of the C-terminal IDR and each residue of the designed peptide^29,30^. This procedure was repeated to obtain a peptide sequence with 10 residues, and the total binding energy was calculated by summing the binding energies. Complementary peptides with the first and second minimal total energies (DP_p53C_2 and DP_p53C_1) were selected among those designed for different initial residues of the C-terminal IDR (Supplementary Table S2). Note that the relative affinities of DP_p53N_ and DP_p53C1_ to the N-terminal and C-terminal peptides of p53 (*K*_D_ = 130 nM for DP_p53N_ and 10 μM for DP_p53C1_) were in line with the estimated contact energies. In this study, we connected two complementary peptides via GSGS- or (GGGS)_3_-linker by taking into account two following points: electrostatic and cation-π interactions between PS residue pairs (e.g. R and D, R and W) were formed between the complementary peptides, and the absolute net charge of the whole PS peptide candidates was ~ 0 for minimizing the electrostatic repulsion. Similarly, complementary peptides, DP_FUS4_ and DP_FUS510_, were designed from 4th–13th residues and 510th–519th residues of FUS, and were then connected by GSGS linker.

### Sequence analysis

The sequences of the designed peptides were compared using the basic local alignment search tool (BLAST)^49^ with the non-redundant (NR) database containing 434,998,385 protein sequences downloaded from the NCBI website in November 2021. We selected the best-matched protein with the highest similarity score to the query peptide. The same procedure was applied to the fragments of the designed peptide sequences DP_p53N_, D7, DP_p53C_1, and DP_p53C_2.

### Peptide samples

Designed peptides and their fragments (DP_p53N_, GSGS-D7, and GSGS-DP_p53C_1) were synthesized using the standard Fmoc-based solid-phase peptide synthesis using the Fmoc-AA-Wang-PEG resins (Watanabe Chemical Industries, Ltd.). The designed peptides were labeled with Alexa488 (Thermo Fisher Scientific) after cleavage from the resin. DP_p53N_ was labeled with 5-FAM (Tokyo Chemical Industry Co., Ltd.) in the presence of N,N’-diisopropylcarbodiimide and 1-hydroxybenzotriazole in dimethylformamide on the resin. The dye-labeled peptides were protected from light to avoid photodegradation. The peptides were cleaved from the resin in the presence of trifluoroacetic acid/water/1,2-ethanedithiol/triisopropylsilane (92.5/2.5/2.5/2.5) for three hours. They were purified by reverse-phase HPLC (RP-HPLC) on a C18 HPLC column using a gradient of water to acetonitrile containing 0.1% trifluoroacetic acid. The purity and identity of each peptide were verified using HPLC and mass spectrometry (Autoflex-T1, Bruker Daltonics). For recruitment measurements into peptide droplets, 5-FAM-labeled peptides of p53 and FUS and N-terminal shuffle peptides of p53 without caps were purchased from Toray Research Center Inc. or SCRUM Inc.

### p53 and FUS samples

We prepared p53 tetramer, dimer, and monomer mutants, as described previously^8,37,50^. For the p53 tetramer, a thermostable and cysteine-modified human p53 mutant (C124A, C135V, C141V, W146Y, C182S, V203A, R209P, C229Y, H233Y, Y234F, N235K, Y236F, T253V, N268D, C275A, C277A, and K292C) was used^50^. The dimer and monomer mutants of p53 corresponded to L344A and L344P of the tetramer sequence, respectively^8,37^. These p53 mutants were expressed with a glutathione S-transferase (GST) tag in *E. coli* and purified after cleavage of the GST tag, as described previously^50,51^. The oligomeric state of p53 mutants was confirmed using a gel filtration column (Superdex 200; GE Healthcare). p53 mutants were labeled with Atto488 (ATTO-TEC) or Alexa488 (Thermo Fisher) using maleimide chemistry and then purified using a heparin column (GE Healthcare). We prepared Alexa488-labeled FUS-MBP as described previously^37,39^.

### DIC and fluorescence microscopy

The DIC and fluorescence modes of an inverted microscope (IX-73; Olympus, Tokyo, Japan) equipped with microscopic objectives (60 × and 10 ×) and a camera (DP74) were used, as described previously^30^. We used the solution containing 0.9–1.8 mM designed peptide, 0.1 μM Alexa488-labeled peptide, 25 mM HEPES, and 0.5 mM EDTA at pH 7.0. To detect the cross-β structure, picogreen was added at 25 µM for DP_p53N_-GSGS-DP_p53C_2 or 2.5 µM for others instead of the Alexa488-labeled peptide. For fusion measurements, we used a solution containing 1 mM designed peptides, 25 mM HEPES, and 0.5 mM EDTA at pH 7.0. For examining the co-phase separation function of designed peptide droplets in the condition of gel-like aggregate formation of p53, we used the solution containing 1 mM designed peptide, 0.1 µM Alexa488-labeled designed peptide, 1.25 or 12.5 µM p53, 0.1 µM AttoRho3b-labeled p53, 20 mM MES, 0.5 mM EDTA, 45 mM NaCl, and 1 mM DTT at pH 5.5. The solutions were incubated at 20 °C for four minutes before casting. The sample solutions were cast onto a coverslip (Matsunami Glass) and covered with a glass slide (Matsunami Glass). The coverslip and slide glass were cleaned with ethanol and 5 M KOH before use. To prevent melting of the designed peptide droplets on the glass surface, we coated the coverslip and slide glass with a 0.5% MPC polymer (Lipidureμ-CM5206; NOF Corp.) in ethanol^36^. DIC and fluorescence images were obtained at 20 or 22 °C. For intrinsic fluorescence measurements, the excitation and detection wavelengths were 440–440 nm and larger than 460 nm, respectively.

### Circular dichroism spectroscopy

We used a circular dichroism spectrometer (J-720; JASCO).

### Single-molecule tracking of designed peptides in droplets

Single-molecule measurements were performed following the method described in our previous studies^37,41^. An inverted fluorescence microscope (IX-73; Olympus) with a total internal reflection fluorescence unit (IX3RFAEVAW; Olympus) was used^52,53^. The objective lens (NA = 1.49) was illuminated using a laser (488 nm) with highly inclined thin illumination (HILO) geometry. The fluorescence collected via the objective lens was detected using an EM-CCD camera (iXon Ultra 888, Andor). The laser power was in the range of 5–30 mW. We used solutions containing 10–100 pM Alexa488-labeled peptide or 10 nM Atto488-labeled p53, 0.9 mM non-labeled peptide or 12.5 μM non-labeled p53, 25 mM HEPES, 0.5 mM EDTA, 45 mM NaCl, and 150 mg/mL dextran at pH 7.0. The sample solutions were cast on a coverslip and covered with a glass slide (Matsunami Glass) using a double-sided tape with a thickness of 5 μm. The coverslip was cleaned with a solution containing 30% H_2_O_2_, 28% NH_3_, and H_2_O in a 1:1:1 ratio before use. The coverslip was coated with 0.5% MPC polymer in ethanol^36^. Images were recorded at intervals of 15–154 ms after reducing the number of observable molecules in the droplets by photobleaching within one minute. The fluorescent spots of single molecules were tracked from sequential images using the ImageJ software with the plugin ‘Particle track and analysis’. We selected trajectories with at least six consecutive points, and MSDs were calculated from all pairs of two-dimensional positions of a molecule at each interval for all trajectories using our in-house program, with some modifications^37,41,53,54^. Average *D* values were calculated by fitting the MSD plots (five data points) using the following equation:

1 $${\text{MSD}} = 4Dt + A,$$

where *t* and *A* represent the time interval and offset, respectively.

### Recruitment of p53 into the designed peptide droplets

Recruitment evaluations were performed following the method described in our previous studies^37,41^. Solutions containing 0.9–1.8 mM designed peptides, 0.1 µM Alexa488-labeled p53 mutants or Alexa488, 25 mM HEPES, 0.5 mM EDTA, and 1 mM DTT at pH 7.0 were used. The solutions were then incubated at 22 °C for five minutes. The aforementioned microscope was used with HILO illumination. To prevent photo-bleaching of the fluorescent samples, we used a laser power of 0.15 mW. The images were acquired at 22 °C. Using the ImageJ software, we calculated the EI values of the individual droplets as follows:

2 $${\text{EI}} = { }\frac{{I_{{{\text{droplet}}}} }}{{I_{{{\text{solution}}}} }},$$

where *I*_droplet_ and *I*_solution_ represent the average fluorescence intensities of the individual droplets and solutions near the droplets with background substitution, respectively.

### Scattering measurements

We detected light scattering corresponding to the designed peptide droplets by measuring OD_350_ values using an absorbance spectrometer (NanoDrop One; Thermo Fisher).

### Titration experiments

The fluorescence anisotropy of the 5-FAM-labled DP_p53N_ was measured with increasing the concentration of GSGS-D7 or GSGS-DP_p53C_1 at 25 °C using a fluorescence spectrometer (FP-6500; JASCO Co., Tokyo, Japan) with an automatic titrator and a home-built autorotating polarizer^50^. Non-labeled peptides were titrated into a solution containing 40 nM 5-FAM-labeled DP_p53N_, 25 mM HEPES, 0.5 mM EDTA, 2 mg/mL BSA, and 2 mM Trolox at pH 7.0. Titration curves were fitted using the following equations based on a one-to-one binding model:

3 $$r_{{{\text{obs}}}} = r_{{\text{A}}} \frac{{\left( {c_{{\text{A}}} - c_{{{\text{AB}}}} } \right)}}{{c_{{\text{A}}} }} + r_{{{\text{AB}}}} \frac{{c_{{{\text{AB}}}} }}{{c_{{\text{A}}} }},$$

4 $$c_{AB} = \frac{{\left( {c_{{\text{A}}} + c_{{\text{B}}} + K_{D} } \right) - \sqrt {\left( {c_{{\text{A}}} + c_{{\text{B}}} + K_{D} } \right)^{2} - 4c_{{\text{A}}} c_{{\text{B}}} } }}{2},$$

where *r*_obs_, *r*_A_, *r*_AB_, *K*_D_, *c*_A_, and *c*_B_ indicate the observed anisotropy, anisotropy of free molecule A, anisotropy of the complex of molecules A and B, dissociation constant, the total concentration of molecule A, and total concentration of molecule B, respectively. The analysis was performed using the Igor software.

## Supplementary Information

Supplementary Information.

## Data Availability

All data generated or analyzed during this study are included in this published article and its Supplementary Information.

## References

[1] Banani SF, Lee HO, Hyman AA, Rosen MK (2017). Biomolecular condensates: Organizers of cellular biochemistry. Nat. Rev. Mol. Cell Biol..

[2] Shin Y, Brangwynne CP (2017). Liquid phase condensation in cell physiology and disease. Science.

[3] Darling AL, Liu Y, Oldfield CJ, Uversky VN (2018). Intrinsically disordered proteome of human membrane-less organelles. Proteomics.

[4] Larson AG, Narlikar GJ (2018). The role of phase separation in heterochromatin formation, function, and regulation. Biochemistry.

[5] Feng Z, Chen X, Wu X, Zhang M (2019). Formation of biological condensates via phase separation: Characteristics, analytical methods, and physiological implications. J. Biol. Chem..

[6] Wang J (2018). A molecular grammar governing the driving forces for phase separation of prion-like RNA binding Proteins. Cell.

[7] Elbaum-Garfinkle S (2015). The disordered P granule protein LAF-1 drives phase separation into droplets with tunable viscosity and dynamics. Proc. Natl. Acad. Sci. U. S. A..

[8] Kamagata K (2020). Liquid-like droplet formation by tumor suppressor p53 induced by multivalent electrostatic interactions between two disordered domains. Sci. Rep..

[9] Hardenberg M, Horvath A, Ambrus V, Fuxreiter M, Vendruscolo M (2020). Widespread occurrence of the droplet state of proteins in the human proteome. Proc. Natl. Acad. Sci. U. S. A..

[10] Brady JP (2017). Structural and hydrodynamic properties of an intrinsically disordered region of a germ cell-specific protein on phase separation. Proc. Natl. Acad. Sci. U. S. A..

[11] Vernon RM (2018). Pi-pi contacts are an overlooked protein feature relevant to phase separation. Elife.

[12] Murthy AC (2019). Molecular interactions underlying liquid-liquid phase separation of the FUS low-complexity domain. Nat. Struct. Mol. Biol..

[13] Krainer G (2021). Reentrant liquid condensate phase of proteins is stabilized by hydrophobic and non-ionic interactions. Nat. Commun..

[14] Borcherds W, Bremer A, Borgia MB, Mittag T (2021). How do intrinsically disordered protein regions encode a driving force for liquid-liquid phase separation?. Curr. Opin. Struct. Biol..

[15] Nakamura H (2018). Intracellular production of hydrogels and synthetic RNA granules by multivalent molecular interactions. Nat. Mater..

[16] Schuster BS (2018). Controllable protein phase separation and modular recruitment to form responsive membraneless organelles. Nat. Commun..

[17] Shin Y (2018). Liquid nuclear condensates mechanically sense and restructure the genome. Cell.

[18] Yoshikawa M, Yoshii T, Ikuta M, Tsukiji S (2021). Synthetic protein condensates that inducibly recruit and release protein activity in living cells. J. Am. Chem. Soc..

[19] Li M (2022). Controlling synthetic membraneless organelles by a red-light-dependent singlet oxygen-generating protein. Nat. Commun..

[20] Abbas M, Lipiński WP, Wang J, Spruijt E (2021). Peptide-based coacervates as biomimetic protocells. Chem. Soc. Rev..

[21] Abbas M, Lipiński WP, Nakashima KK, Huck WTS, Spruijt E (2021). A short peptide synthon for liquid-liquid phase separation. Nat. Chem..

[22] Sun Y (2022). Phase-separating peptides for direct cytosolic delivery and redox-activated release of macromolecular therapeutics. Nat. Chem..

[23] Gabryelczyk B (2019). Hydrogen bond guidance and aromatic stacking drive liquid-liquid phase separation of intrinsically disordered histidine-rich peptides. Nat. Commun..

[24] Hughes MP (2018). Atomic structures of low-complexity protein segments reveal kinked β sheets that assemble networks. Science.

[25] Luo F (2018). Atomic structures of FUS LC domain segments reveal bases for reversible amyloid fibril formation. Nat. Struct. Mol. Biol..

[26] Gui X (2019). Structural basis for reversible amyloids of hnRNPA1 elucidates their role in stress granule assembly. Nat. Commun..

[27] Wei W (2016). An underwater surface-drying peptide inspired by a mussel adhesive protein. Adv. Funct. Mater..

[28] Yuan CQ (2019). Nucleation and growth of amino acid and peptide supramolecular polymers through liquid-liquid phase separation. Angew. Chem. Int. Ed..

[29] Kamagata K (2019). Rational design using sequence information only produces a peptide that binds to the intrinsically disordered region of p53. Sci. Rep..

[30] Kamagata K (2022). Rational peptide design for regulating liquid-liquid phase separation on the basis of residue-residue contact energy. Sci. Rep..

[31] Nott TJ (2015). Phase transition of a disordered nuage protein generates environmentally responsive membraneless organelles. Mol. Cell.

[32] Das S, Eisen A, Lin YH, Chan HS (2018). A lattice model of charge-pattern-dependent polyampholyte phase separation. J. Phys. Chem. B.

[33] Hazra MK, Levy Y (2020). Charge pattern affects the structure and dynamics of polyampholyte condensates. Phys. Chem. Chem. Phys..

[34] Martin EW (2020). Valence and patterning of aromatic residues determine the phase behavior of prion-like domains. Science.

[35] Yamazaki H, Takagi M, Kosako H, Hirano T, Yoshimura SH (2022). Cell cycle-specific phase separation regulated by protein charge blockiness. Nat. Cell Biol..

[36] Igarashi C (2017). DNA garden: A simple method for producing arrays of stretchable DNA for single-molecule fluorescence imaging of DNA-binding proteins. Bull. Chem. Soc. Jpn..

[37] Kamagata K (2021). Molecular principles of recruitment and dynamics of guest proteins in liquid droplets. Sci. Rep..

[38] Mora AK, Singh PK, Patro BS, Nath S (2016). PicoGreen: A better amyloid probe than Thioflavin-T. Chem. Commun..

[39] Kamagata K (2021). Characterization of design grammar of peptides for regulating liquid droplets and aggregates of FUS. Sci. Rep..

[40] Chan FT (2013). Protein amyloids develop an intrinsic fluorescence signature during aggregation. Analyst.

[41] Kamagata K (2022). Structure-dependent recruitment and diffusion of guest proteins in liquid droplets of FUS. Sci. Rep..

[42] Nott TJ, Craggs TD, Baldwin AJ (2016). Membraneless organelles can melt nucleic acid duplexes and act as biomolecular filters. Nat. Chem..

[43] Wei MT (2017). Phase behaviour of disordered proteins underlying low density and high permeability of liquid organelles. Nat. Chem..

[44] Qamar S (2018). FUS phase separation is modulated by a molecular chaperone and methylation of arginine cation-pi interactions. Cell.

[45] Cakmak FP, Choi S, Meyer MO, Bevilacqua PC, Keating CD (2020). Prebiotically-relevant low polyion multivalency can improve functionality of membraneless compartments. Nat Commun.

[46] Cinar H (2019). Temperature, hydrostatic pressure, and osmolyte effects on liquid-liquid phase separation in protein condensates: physical chemistry and biological implications. Chem. Eur. J..

[47] Li S (2021). Pressure and temperature phase diagram for liquid-liquid phase separation of the RNA-binding protein fused in sarcoma. J. Phys. Chem. B.

[48] Miyazawa S, Jernigan RL (1996). Residue-residue potentials with a favorable contact pair term and an unfavorable high packing density term, for simulation and threading. J. Mol. Biol..

[49] Altschul SF, Gish W, Miller W, Myers EW, Lipman DJ (1990). Basic local alignment search tool. J. Mol. Biol..

[50] Murata A (2015). One-dimensional sliding of p53 along DNA is accelerated in the presence of Ca^2+^ or Mg^2+^ at millimolar concentrations. J. Mol. Biol..

[51] Murata A (2017). One-dimensional search dynamics of tumor suppressor p53 regulated by a disordered C-terminal domain. Biophys. J..

[52] Kamagata K, Mano E, Ouchi K, Kanbayashi S, Johnson RC (2018). High free-energy barrier of 1D diffusion along DNA by architectural DNA-binding proteins. J. Mol. Biol..

[53] Kamagata K (2020). The HMGB chromatin protein Nhp6A can bypass obstacles when traveling on DNA. Nucleic Acids Res..

[54] Kamagata K (2021). Testing mechanisms of DNA sliding by architectural DNA-binding proteins: Dynamics of single wild-type and mutant protein molecules in vitro and in vivo. Nucleic Acids Res..

