# Supplementary material for: Rational design of phase separating peptides based on phase separating protein sequence of p53
Source: Sci Rep. 2023 Apr 6;13:5648. doi: 10.1038/s41598-023-32632-2 (PMC10079954; doi:10.1038/s41598-023-32632-2)
Supplement: Supplementary file 1 — Supplementary Information. [file 41598_2023_32632_MOESM1_ESM.docx]

Supplementary Information for

Rational design of phase separating peptides based on phase separating protein sequence of p53

*Corresponding author: Kiyoto Kamagata

Institute of Multidisciplinary Research for Advanced Materials, Tohoku University, Katahira 2-1-1, Aoba-ku, Sendai 980-8577, Japan

TEL: +81-22-217-5843/FAX: +81-22-217-5842

e-mail: kiyoto.kamagata.e8@tohoku.ac.jp


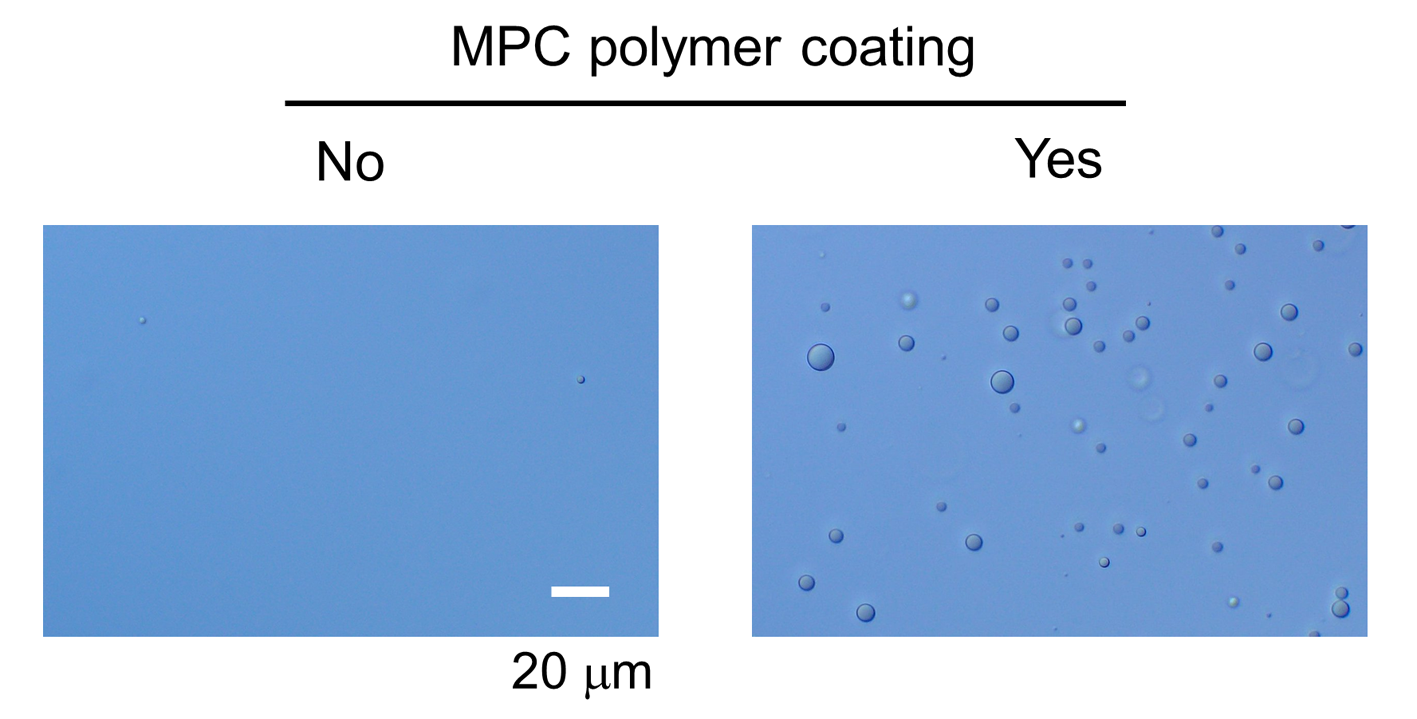


**Fig. S1 Coating with the MPC polymer enabled the observation of the peptide droplets using DIC microscopy.** DIC images of DP_p53N_-GSGS-D7 (2 mM) in the presence and absence of MPC polymer coating on the coverslip and slide glass. The measurements were conducted in a solution containing 25 mM HEPES and 0.5 mM EDTA at pH 7.0 and 22 °C.


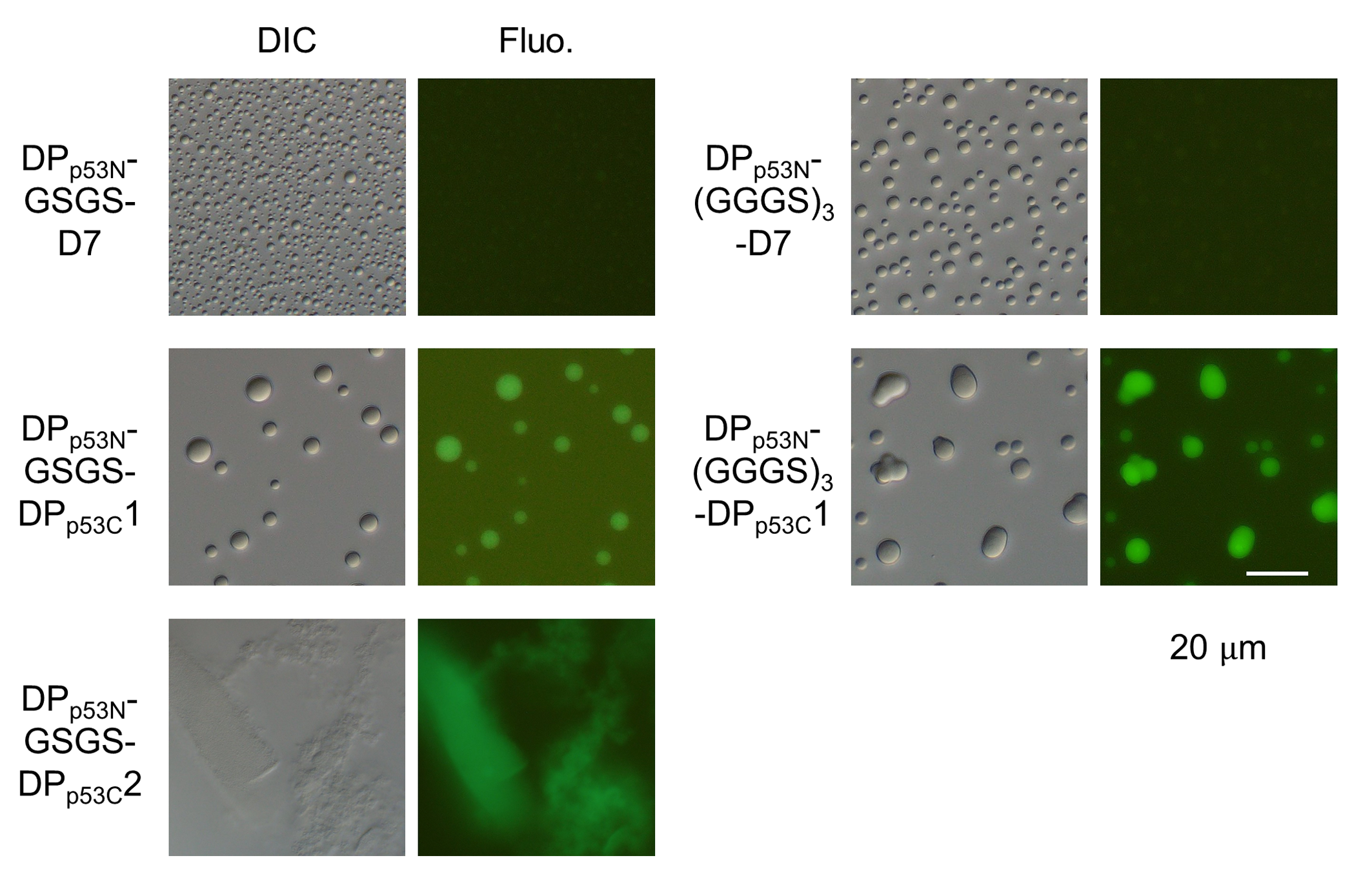


**Fig. S2 Measurements of intrinsic fluorescence from droplets or aggregates of designed peptides.** DIC and fluorescence images of the solution containing peptides. The measurements were conducted in a solution containing 1 mM peptides, 25 mM HEPES, and 0.5 mM EDTA at pH 7.0 and 20 °C after incubation for 1 hour. The fluorescence upon 400~440 nm excitation represents the intrinsic one reflecting amyloid structure.


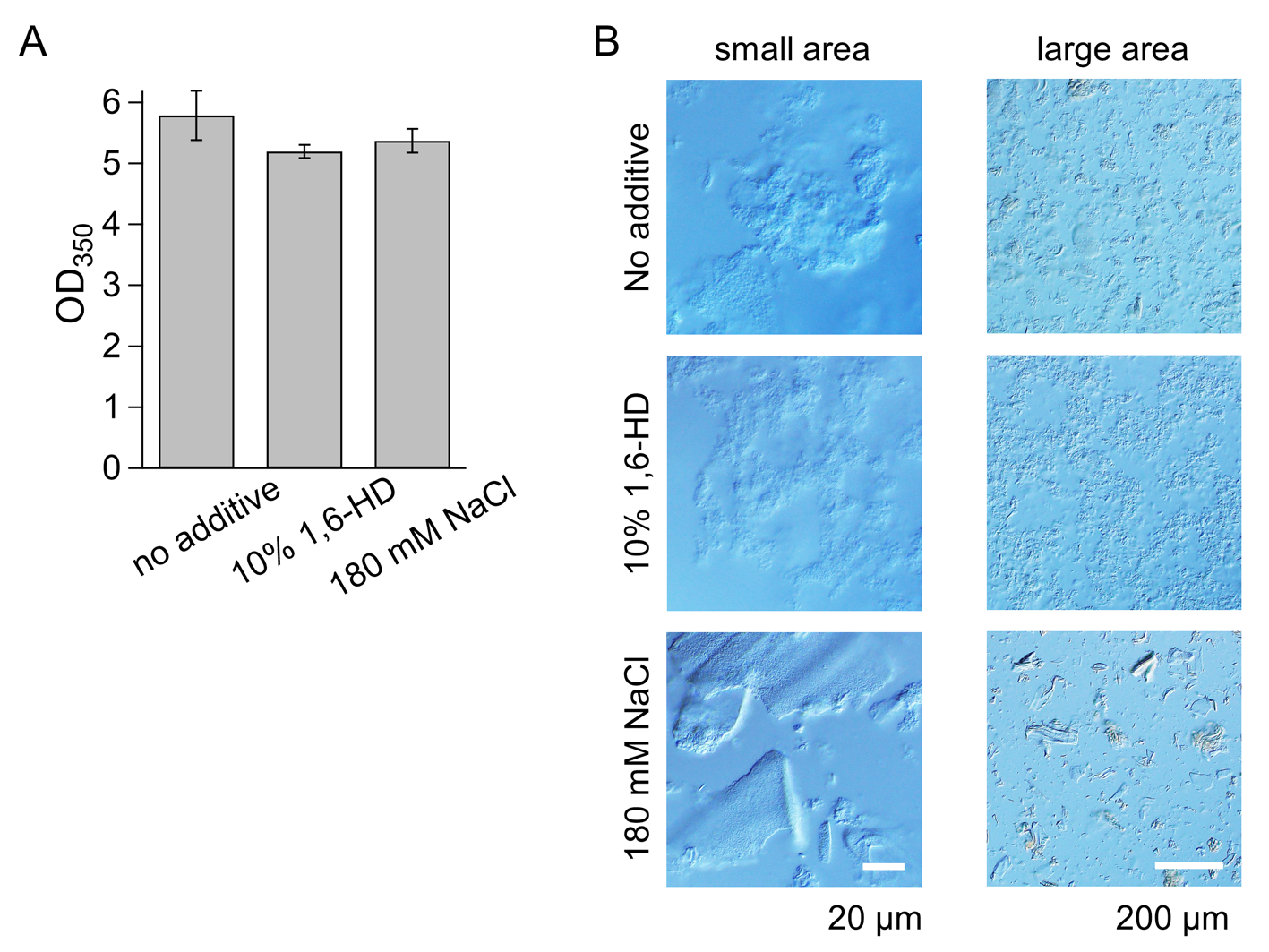


**Fig. S3 Formation of solid aggregates of DP_p53N_-GSGS-DP_p53C_2 was not significantly affected by the addition of 1,6-hexanediol and salt.** (A, B) Effect of additives, 1,6-hexandiol (1,6-HD) and NaCl, on aggregate formation of DP_p53N_-GSGS-DP_p53C_2 was monitored as the scattering intensity observed at 350 nm (A) and via DIC images (B). The measurements were conducted in a solution containing 25 mM HEPES, 0.5 mM EDTA, and 1 mM DP_p53N_-GSGS-DP_p53C_2 in the presence or absence of additives at pH 7.0 and 22 °C.


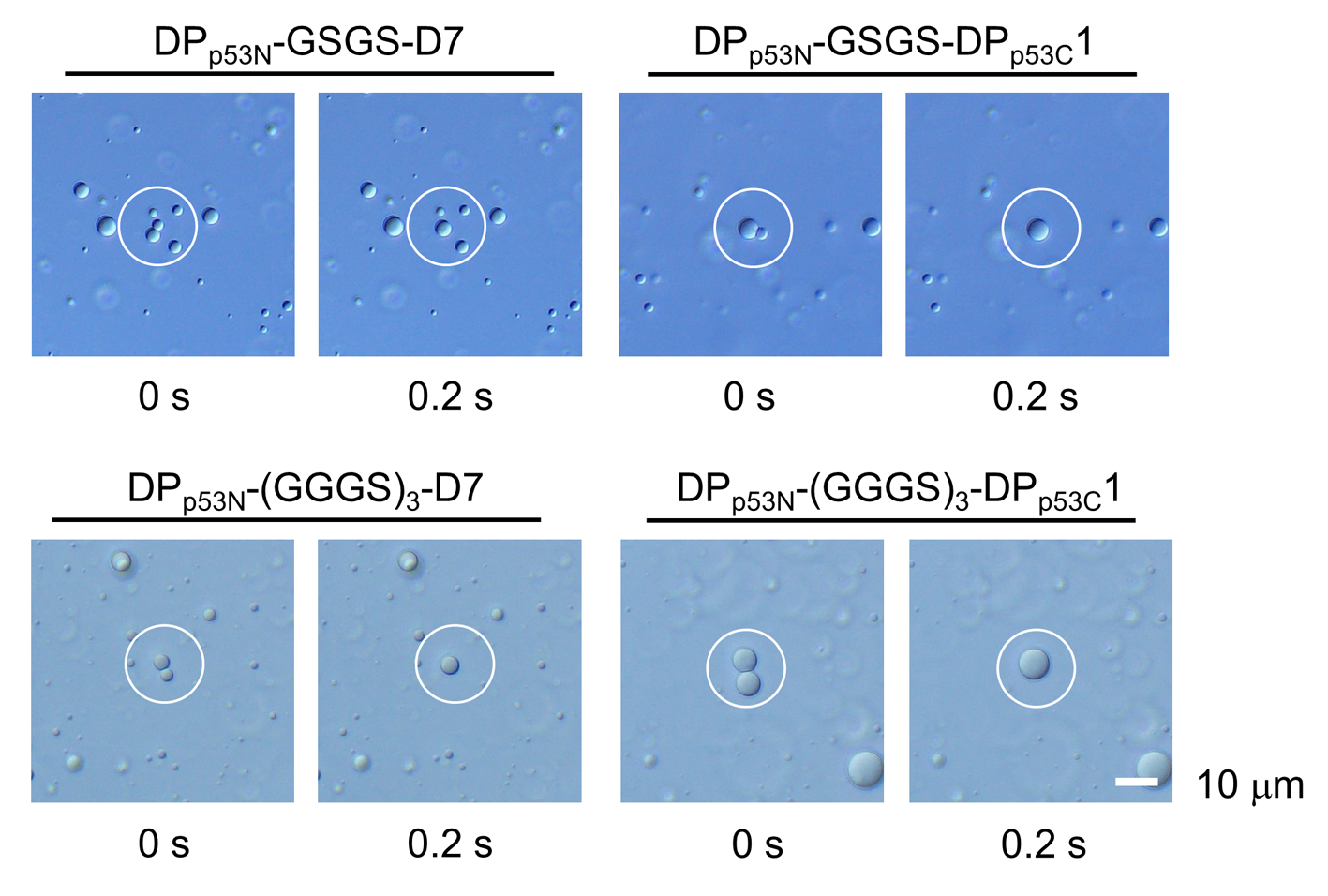


**Fig. S4 Two droplets of designed peptides fused within 0.2 s under the conditions involving p53 droplet fusion.** Typical fusion snapshots of two droplets (white circles) observed in DIC images of the solution containing 1 mM designed peptides, 25 mM HEPES, 0.5 mM EDTA, 45 mM NaCl, and 150 mg/mL dextran at pH 7.0 and 22 °C.


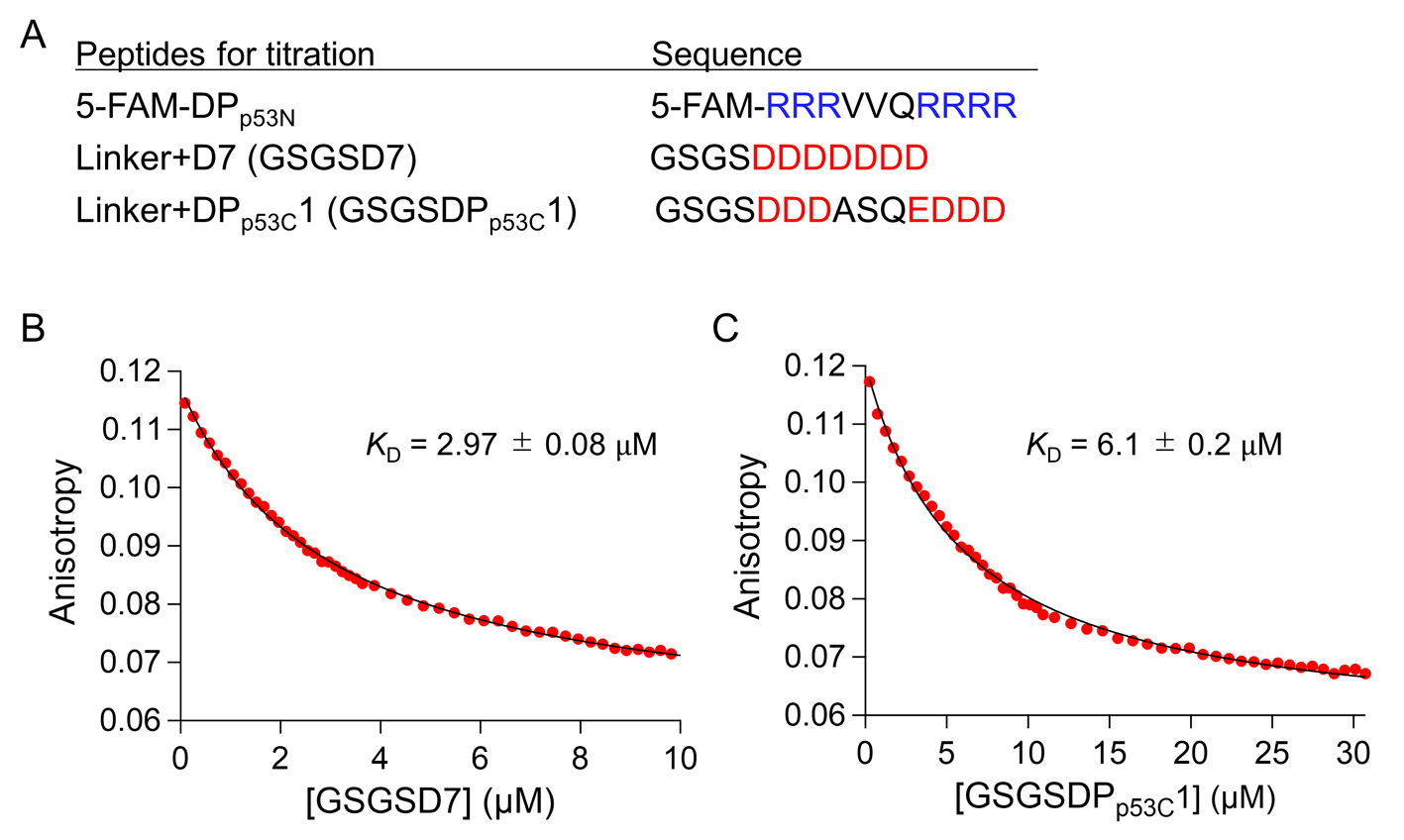


**Fig. S5 DP_p53N_ binds to D7 more strongly than to DP_p53C_1.** (A) Designed peptide fragment sequences used for titration measurements. (B, C) Changes in the fluorescence anisotropy for the association between the designed peptide fragments. In panels (B) and (C), the solid curves denote the best-fitted curve based on the equation assuming one-to-one binding ^1^, and the *K*_D_ values obtained from the fitting are shown.


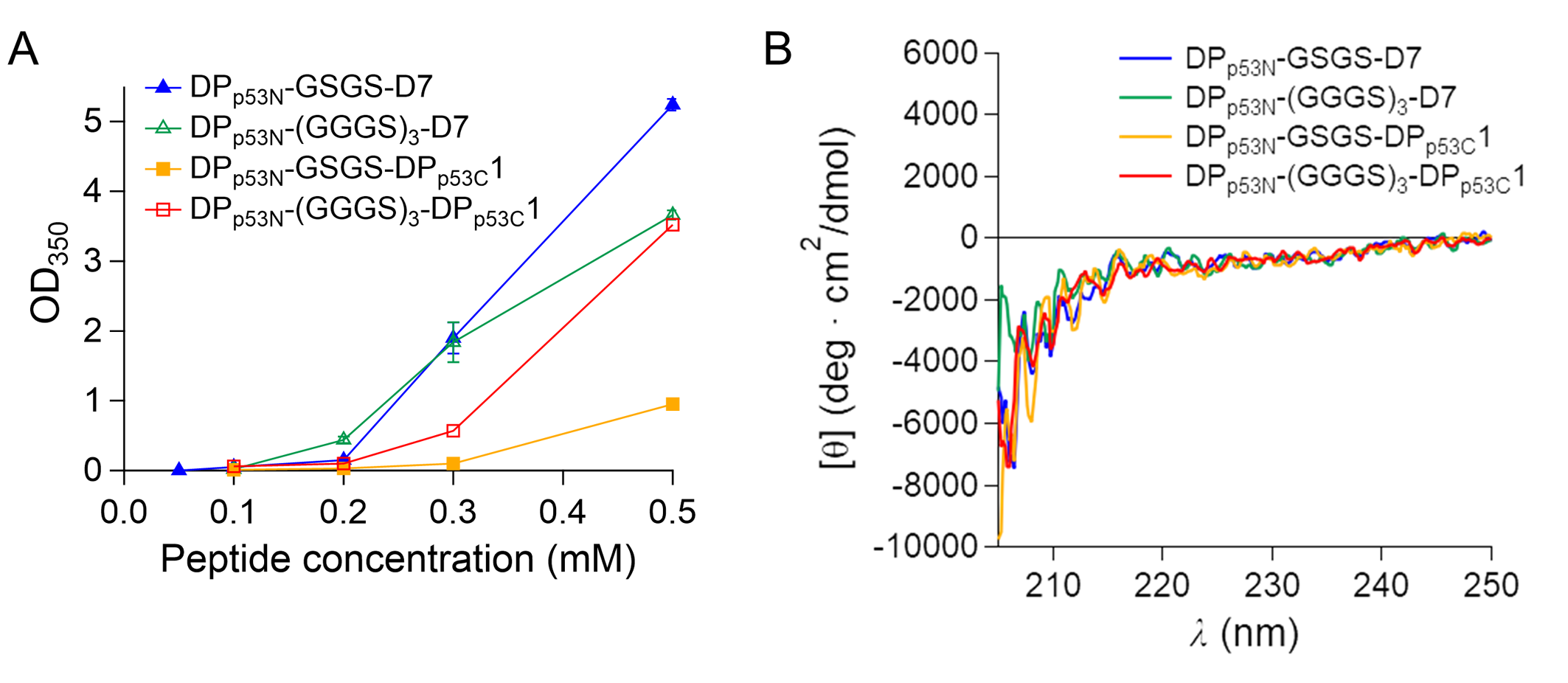


**Fig. S6 Critical concentration of designed peptides are 100~200 µM and peptides** **are in random coil states under no phase separating condition.** (A) Dependence of the designed peptide concentrations on the droplet formation. The scattering intensities at 350 nm in 104 mM Hepes and 0.5 mM EDTA (pH 7.0) were presented at 22 °C. Error bars denote the standard errors of three measurements. (B) Circular dichroism spectra of designed peptides under no phase separating condition. The spectra were taken in a solution containing 0.1 mg/mL designed peptides, 25 mM HEPES, and 0.5 mM EDTA at pH 7.0 and 26.6 °C.


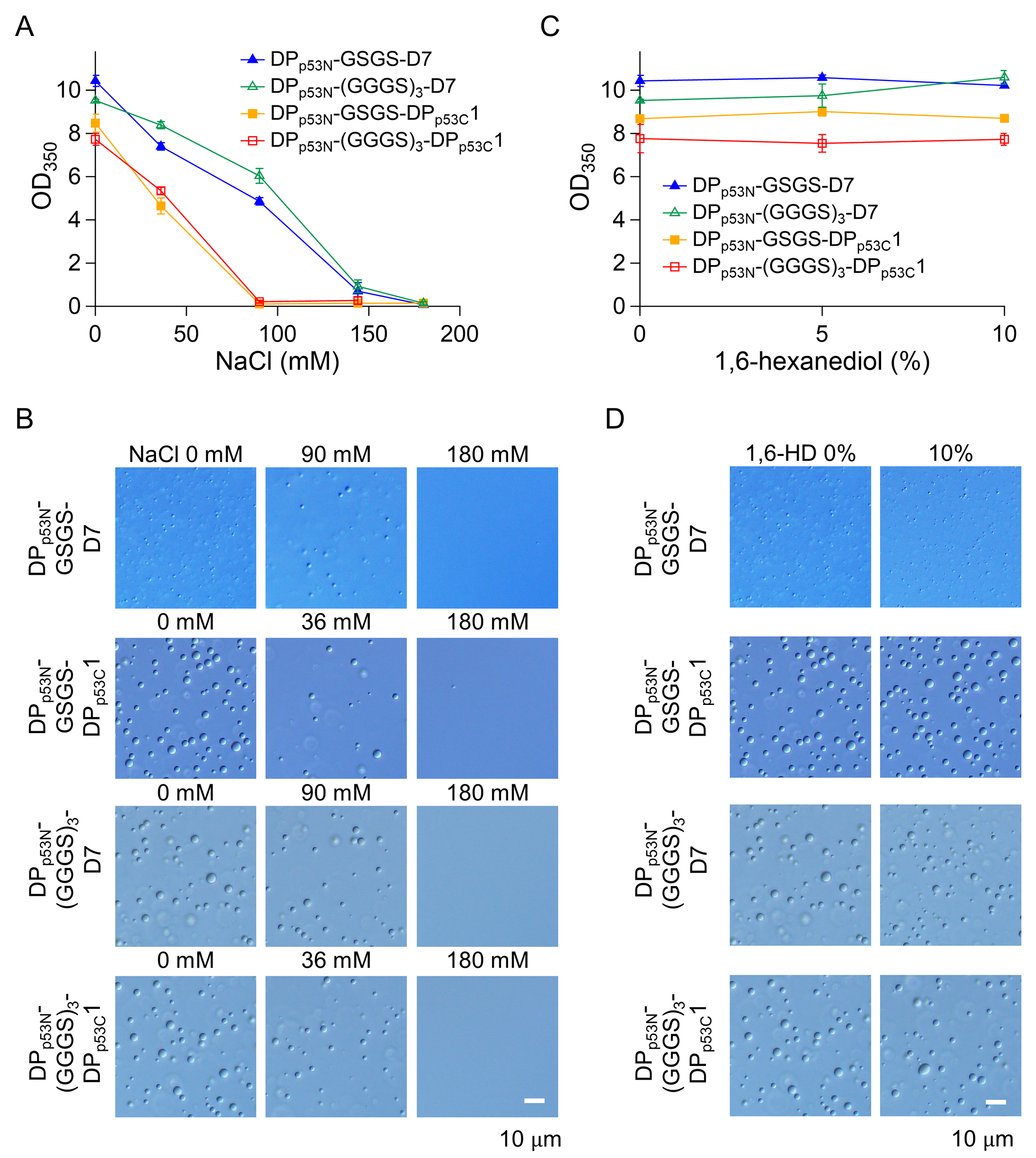


**Fig. S7** **Designed peptide droplets are sensitive to salt concentration but not to 1,6-hexandiol.** (A, B) Salt-concentration dependence of droplet formation of designed peptides monitored as the scattering intensity at 350 nm (A) and via DIC images (B). (C, D) Effect of 1,6-hexandiol on droplet formation of designed peptides monitored as the scattering intensity at 350 nm (C) and via DIC images (D). The measurements were performed in a solution containing 1 mM designed peptides, 25 mM HEPES, and 0.5 mM EDTA at pH 7.0 and 22 °C. In panels (A) and (C), the errors denote the standard errors of at least three measurements.


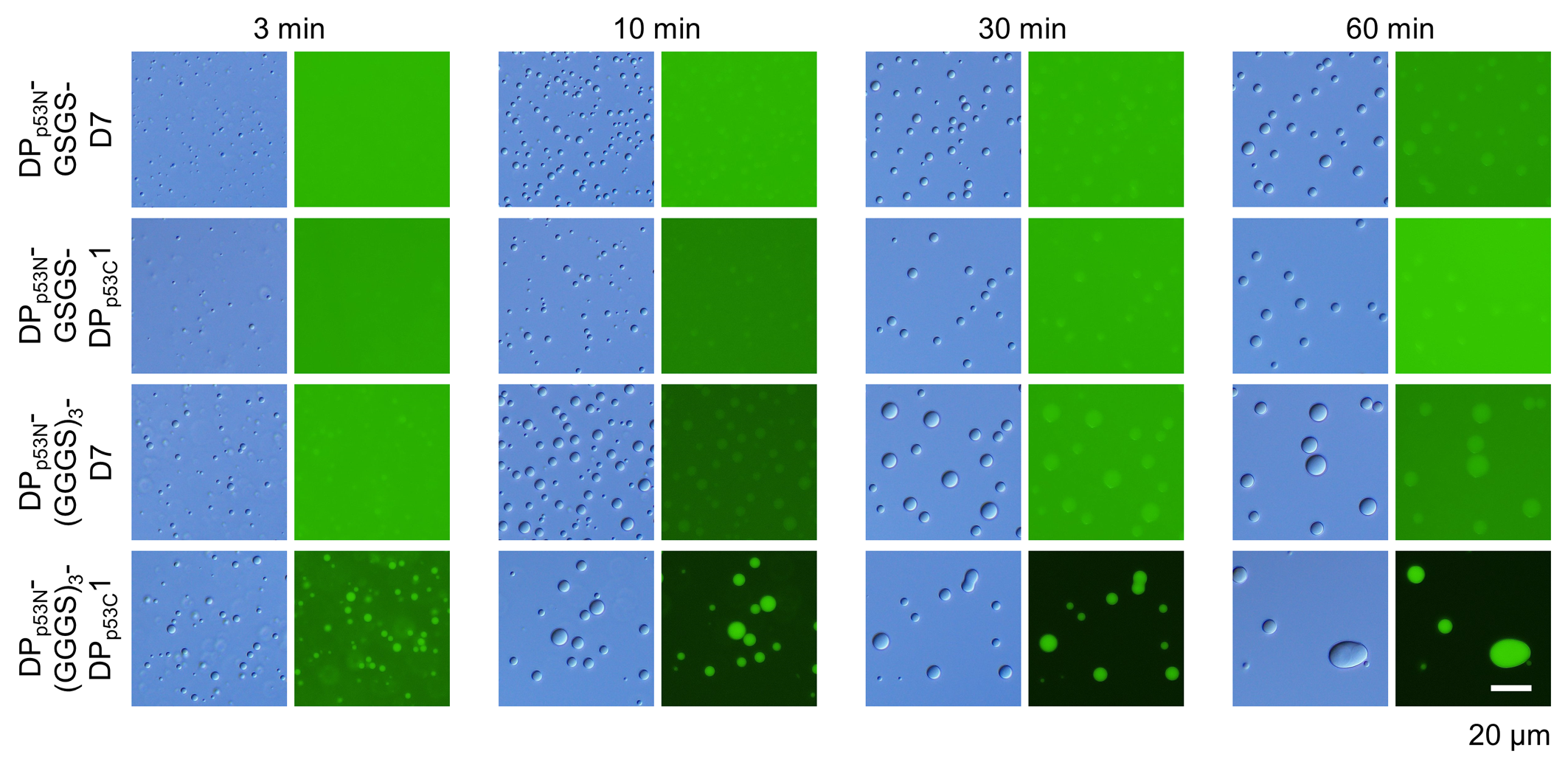


**Fig. S8** **Time course of cross-β structure formation of designed peptides in droplets monitored by DIC and fluorescence images.** The fluorescence was detected corresponding to the amyloid marker Picogreen at 2.5 µM in a solution containing 0.9 mM designed peptides, 25 mM HEPES, and 0.5 mM EDTA at pH 7.0 and 22 °C.


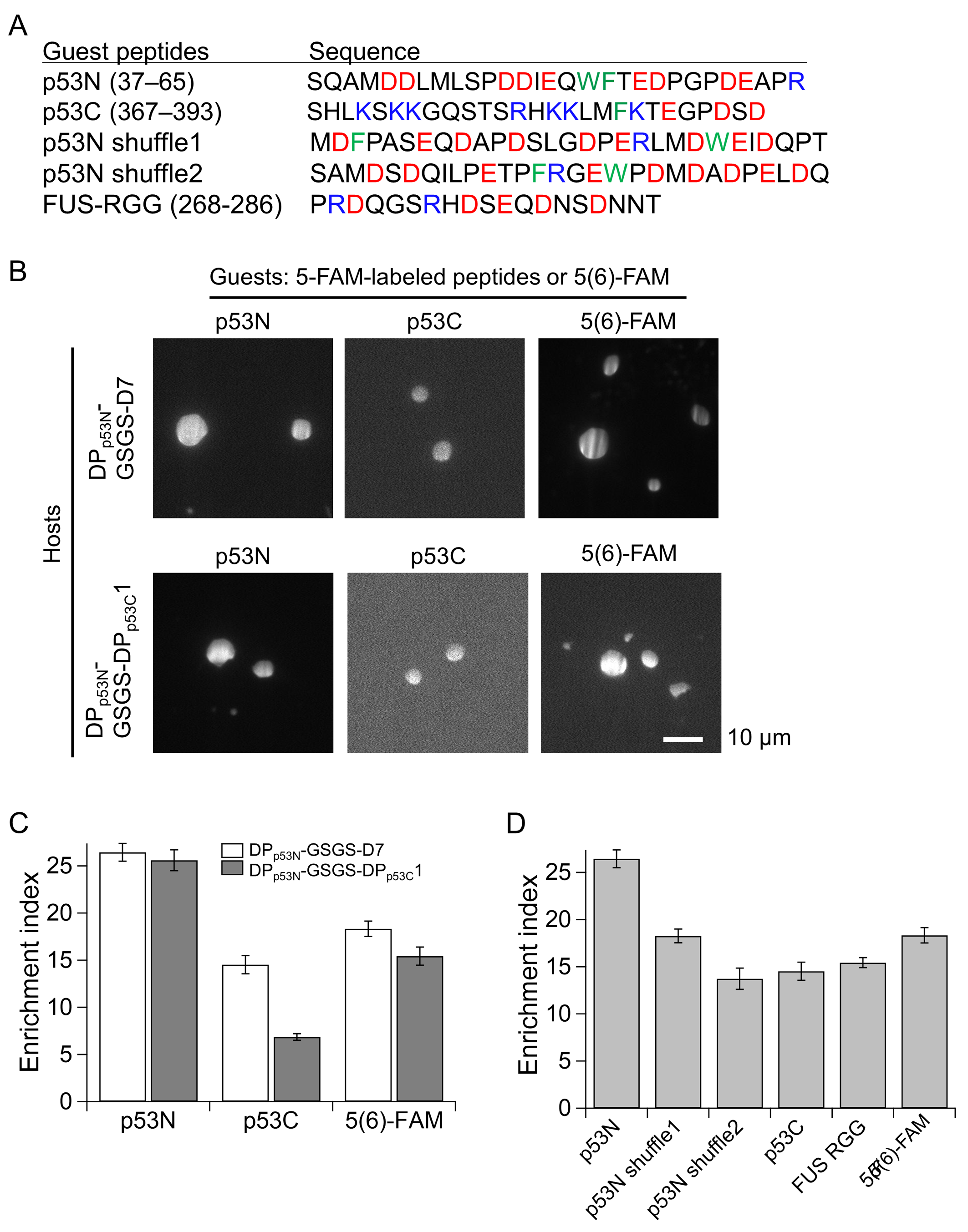


**Fig. S9 N-terminal peptide of p53 is recruited in designed peptide droplets more frequently than C-terminal peptide of p53.** (A) Sequences of N-terminal and C-terminal peptides of p53 (p53N and p53C), N-terminal shuffle peptides of p53 (p53N shuffle1 and 2), and peptide of RGG domain of FUS (FUS-RGG). (B) Fluorescence images of FAM-labeled p53 peptides and FAM only in droplet solution of non-labeled designed peptides. The solution containing 25 mM HEPES and 0.5 mM EDTA at pH 7.0 was used. (C) Enrichment index of p53 peptides with respect to the designed peptide droplets. (D) Enrichment index of guest peptides with respect to the droplets of DP_p53N_-GSGS-D7. In panels (C) and (D), error bars denote the standard errors.


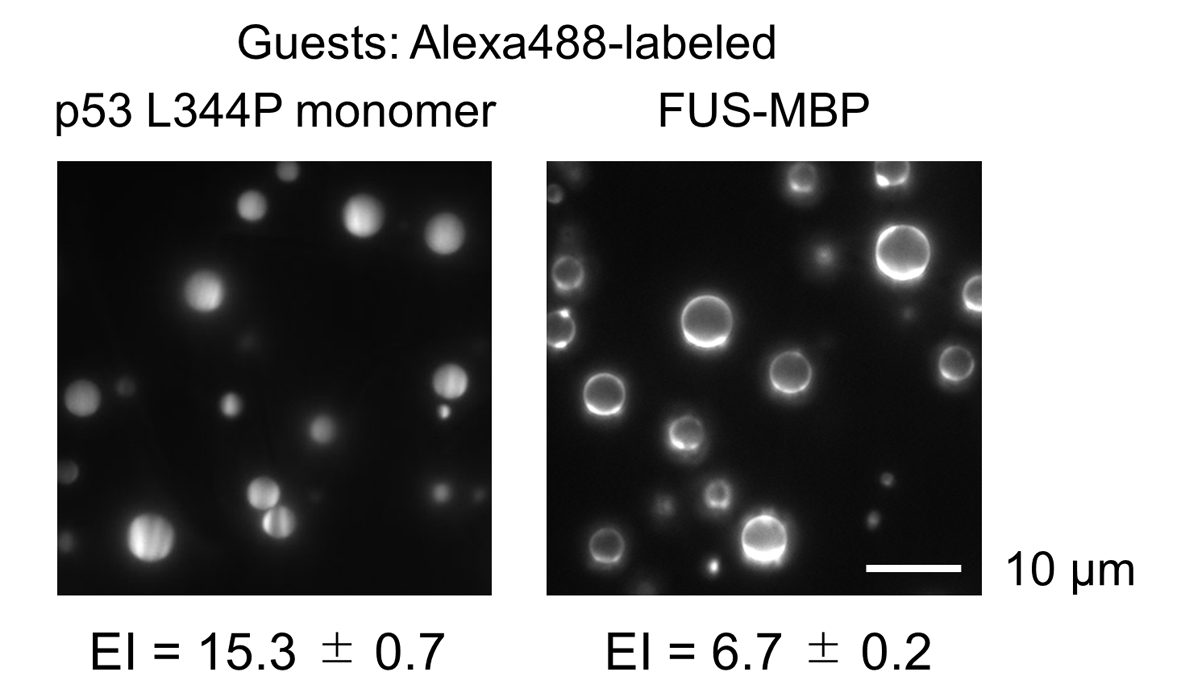


**Fig. S10 p53 monomer is localized inside droplets of DP_p53N_-GSGS-D7, whereas FUS-MBP is localized in its droplet surfaces.** Fluorescence images of Alexa488-labeled p53 monomer mutant (L344P) and FUS-MBP in droplet solution of non-labeled DP_p53N_-GSGS-D7. The solution containing 103 mM HEPES and 1 mM DTT, 100 nM Alexa488 labeled proteins, and 1 mM DP_p53N_-GSGS-D7 at pH 7.0 was used.


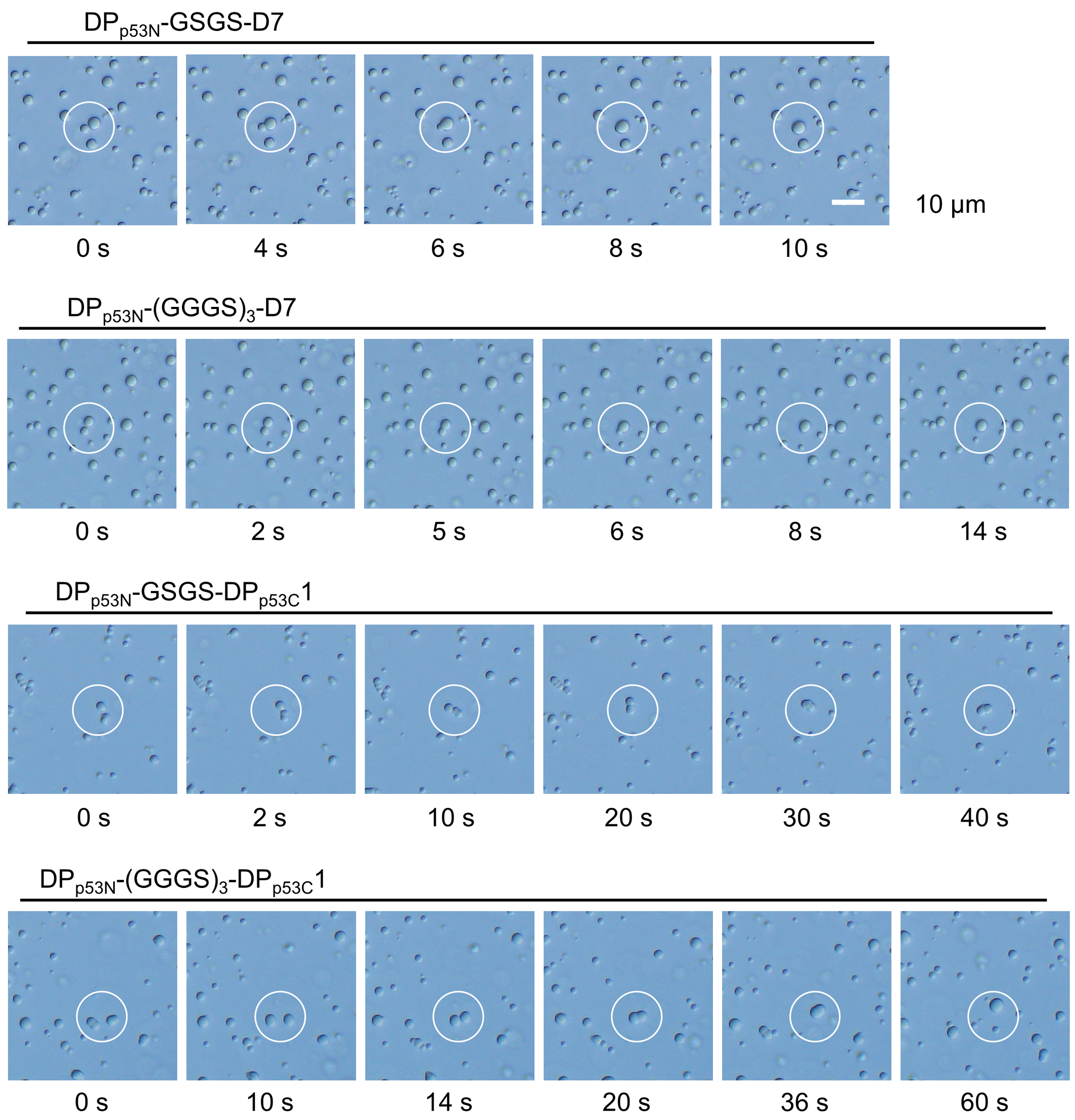


**Fig. S11 DIC microscopy confirmed that the two droplets of designed peptides containing p53 tetramer fused with each other.** Typical fusion snapshots of two droplets (white circles) in the solution containing 20 mM MES, 0.5 mM EDTA, 45 mM NaCl, 1 mM DTT, 1.25 µM p53, 0.1 µM AttoRho3B-labeled p53, 1 mM designed peptide, and 0.1 µM Alexa488-labeled designed peptide at pH 5.5 and 22 °C.


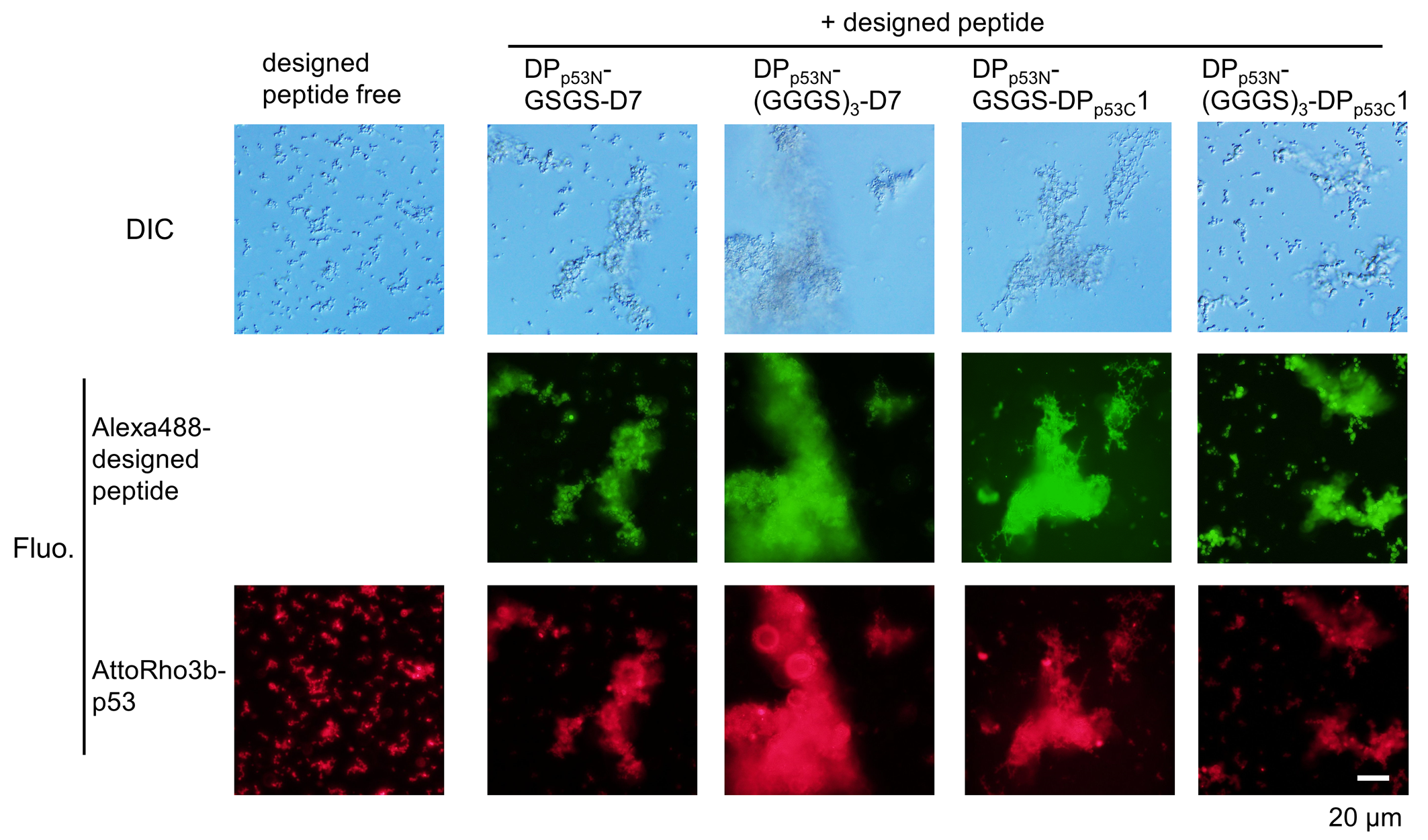


**Fig. S12 Designed peptide droplets did not suppress gel-like aggregate formation of p53 when the mass of the designed peptides was 4.7~6.1-fold against that of p53.** DIC and fluorescence images of the solution containing 1 mM of the designed peptides and 12.5 µM p53 (tetramer) in 45 mM NaCl at pH 5.5. For visualizing the localization, Alexa488-labeled designed peptide and AttoRho3b-labeled p53 were added at a concentration of 0.1 µM.


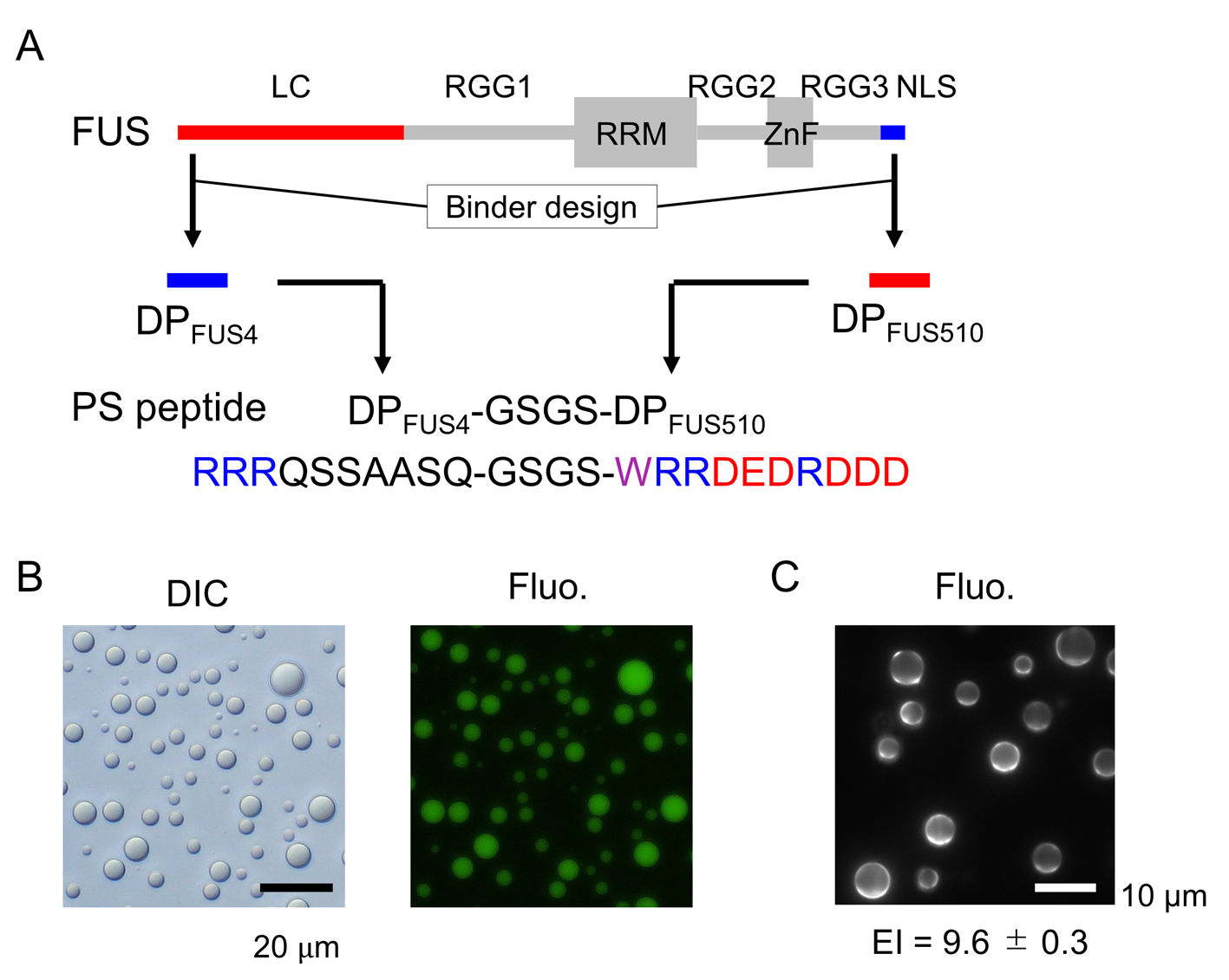


**Fig. S13 Design and experimental verification of artificial PS peptides from PS protein FUS.** (A) The design scheme and peptide sequences designed based on the FUS sequence. LC, RGG1, RRM, RGG2, ZnF, RGG3, and NLS represent individual domains of FUS. Large boxes represent folded regions, whereas thin lines represent disordered regions. Peptide fragments (DP_FUS4_ and DP_FUS510_) were designed based on two IDRs (LC and NLS) of FUS and were then connected by GSGS linker. DP_FUS4_ and DP_FUS510_ represent peptides designed from 4^th^−13^th^ residues and 510^th^−519^th^ residues of FUS, respectively. (B) DIC and fluorescence images of the solution containing 4 mM FUS designed peptide and 120 mM HEPES at pH 7.0. Alexa488-labeled peptide at 0.1 μM was added to confirm high concentrations in liquid droplets. (C) Fluorescence images of Alexa488-labeled FUS-MBP in non-labeled FUS-designed-peptide droplet solution. The solution contained 4 mM FUS designed peptide, 103 mM HEPES, 1 mM DTT, and 0.1 μM Alexa488-labeled FUS-MBP.

**Table S1: Analysis of the sequence identity of designed peptides to natural proteins**

| Sequence | Best matched protein^#^ | Matched region | Sequence of the matched region^*^ | Sequence identity |
| --- | --- | --- | --- | --- |
| RRRVVQRRRR | 6VZI_G | 460-469 | RRRVVQRRRR | 10/10 |
| DDDASQEDDD | ALU57796.1 | 91-100 | DDDASrEDDn | 8/10 |
| DDDDDDD | NXR39938.1 | 2672-2678 | DDDDDDD | 7/7 |
| EDDDWWMWWD | MBL8861226.1 | 210-219 | EDDsWWlWWe | 7/10 |
| RRRVVQRRRRGSGSDDDDDDD | KAG2206627.1 | 962-982 | eedgVkRRRRrSGpDDDnDeD | 12/21 |
| RRRVVQRRRRGSGSDDDASQEDDD | KAG5413522.1 | 194-217 | skvgysRRRRGSGSDDDdStEDae | 14/24 |
| RRRVVQRRRRGSGSEDDDWWMWWD | KAG8988809.1 | 393-416 | vnRlaQRRqRvdGaEDDnqsMWft | 11/24 |
| RRRVVQRRRRGGGSGGGSGGGSDDDDDDD | PIA63024.1 | 1548-1576 | RkRsaQRnsRGGktGpemGGSDeeeDDqe | 12/29 |
| RRRVVQRRRRGGGSGGGSGGGSDDDASQEDDD | WP_094374458.1 | 218-249 | tvlvgagsgkGGtasGGSGpGaDaDApeEDDg | 13/32 |

^#^ Protein is represented as a sequence version number provided by NCBI.

* Identical amino acids are denoted by uppercase letters.

**Table S2: Designed peptide sequences for C-terminal IDR of p53 and their estimated contact energy (one by three design with MD-based energy).**

| p53 sequence number for design (initial residue number) | Estimated contact energy (kJ/mol) | Sequence of designed peptide |
| --- | --- | --- |
| 358 | -26.4 | DRAAEEDAQQ |
| 359 | -25.3 | RAAEEDAQQH |
| 360 | -24.9 | AAEEDAQQHW |
| 361 | -23.5 | AEEDAQQHWE |
| 362 | -25.0 | EEDAQQHWED |
| 363 | -24.5 | EDAQQHWEDD |
| 364 | -23.8 | DAQQHWEDDD |
| 365 | -20.6 | AQQHWEDDDD |
| 366 | -20.8 | QQHWEDDDDA |
| 367 | -21.2 | QHWEDDDDAS |
| 368 | -21.8 | HWEDDDDASQ |
| 369 | -25.9 | WEDDDDASQE |
| 370 | -29.2 | EDDDDASQED |
| 371 | -35.6 | DDDDASQEDD |
| 372 | -36.0 | DDDASQEDDD (DP_p53C1_) |
| 373 | -36.0 | DDASQEDDDW |
| 374 | -34.0 | DASQEDDDWW |
| 375 | -33.3 | ASQEDDDWWM |
| 376 | -34.7 | SQEDDDWWMW |
| 377 | -35.9 | QEDDDWWMWW |
| 378 | -36.4 | EDDDWWMWWD (DP_p53C2_) |
| 379 | -33.4 | DDDWWMWWDR |
| 380 | -30.5 | DDWWMWWDRR |
| 381 | -25.7 | DWWMWWDRRR |
| 382 | -24.8 | WWMWWDRRRR |
| 383 | -30.7 | WMWWDRRRRR |

**References**

1. Murata, A., et al. One-dimensional search dynamics of tumor suppressor p53 regulated by a disordered C-terminal domain. *Biophys. J.* **112**, 2301-2314 (2017).
